# Synergic Effect of Fluconazole and Quinoline Derivatives Combination Against *Cryptococcus* spp., Mechanisms of Action and Toxicity

**DOI:** 10.3390/microorganisms14081654

**Published:** 2026-07-29

**Authors:** Luana Candice Genz Bazana, Ânderson Ramos Carvalho, Rodrigo Foss da Silva, Solange Cristina Garcia, Marcelo Dutra Arbo, Mario Lettieri Teixeira, Alexandre Meneghello Fuentefria

**Affiliations:** 1 Laboratório de Pesquisa em Micologia Aplicada, Universidade Federal do Rio Grande do Sul, Porto Alegre 90620-170, RS, Brazil; luanacgb93@hotmail.com (L.C.G.B.); alexandre.fuentefria@ufrgs.br (A.M.F.); 2 Programa de Pós-Graduação em Ciências Farmacêuticas, Universidade Federal do Rio Grande do Sul, Porto Alegre 90160-093, RS, Brazilmarcelo.arbo@ufrgs.br (M.D.A.); 3 Laboratório de Toxicologia, Universidade Federal do Rio Grande do Sul, Porto Alegre 90620-170, RS, Brazil; 4 Laboratório de Pesquisa em Toxicologia e Farmacologia, Instituto Federal Catarinense, Concórdia 89703-720, SC, Brazil; mario.teixeira@ifc.edu.br

**Keywords:** antifungal combination, cryptococcal infection, *Cryptococcus* spp., checkerboard assay, amphotericin B, fluconazole, metal chelator

## Abstract

Cryptococcosis is a severe fungal infection affecting immunocompromised individuals, with treatment limited to FLZ, AMB, and FC. This study investigated the antifungal potential of fluconazole (FLZ)-, amphotericin B (AMB)-, 8-hydroxyquinoline (8HQ)-, and clioquinol (CQ)-based combinations against *Cryptococcus neoformans* and *C. gattii*. Drug interactions were assessed by checkerboard assay, followed by time–kill curves, irritability, toxicity, and virulence factors inhibition tests. The FLZ + AMB combination showed weak synergism, whereas FLZ combined with CQ or 8HQ exhibited strong synergistic effects (*p* < 0.001) at low concentrations (0.125–0.25 µg/mL), up to three times greater than those of FLZ + AMB. This effect persisted across other strains, including less FLZ-susceptible isolates. Moreover, FLZ + CQ inhibited melanin production in both species without causing significant irritability or toxicity in the tested models. These results indicate that combining drugs with distinct mechanisms of action can enhance antifungal efficacy while potentially reducing treatment doses. The FLZ + CQ/8HQ combinations represent promising candidates for future in vivo models for therapeutic evaluation.

## 1. Introduction

Cryptococcosis is a fungal infection caused by the *Cryptococcus* genus. The most severe form of the disease is cryptococcal meningitis, usually associated with immunocompromised patients. *C. neoformans* is the predominant species isolated in these infections, however species such as *C. gattii* can cause infection in immunocompetent patients [1,2]. *Cryptococcus* spp. is widely distributed from the environment and frequently found in dry pigeon droppings (intermediate host), tree bark, and soil [3].

The spores present in the environment can be inhaled, and when not cleared and eliminated by the respiratory tract defense cells, they can remain dormant in the pulmonary alveoli without presenting symptoms [4]. In immunocompromised individuals, dormant cells can be reactivated and disseminated through the lung tissue, bloodstream and central nervous system, for which it has a predilection, causing cryptococcal meningitis [5].

It is estimated that 181,000 deaths occur per year due to cryptococcal meningitis worldwide, with high mortality rates and high hospitalization costs, representing a public health problem, especially in underdeveloped countries and HIV-endemic regions [6]. According to the guideline for the management of cryptococcal infections published in 2024, the standard therapy for cryptococcal meningitis is either: (1) liposomal Amphotericin B 3–4 mg/kg daily plus flucytosine 25 mg/kg four times a day or (2) a single dose of liposomal Amphotericin B 10 mg/kg, with 2 weeks of flucytosine 25 mg/kg four times a day and fluconazole 1200 mg daily for two weeks [7].

Despite updates to the therapeutic regimen, clinical outcomes for cryptococcosis remain difficult, with high mortality rates [7,8]. Furthermore, currently available antifungal agents have issues crossing the blood–brain barrier, toxicity due to long treatment times, and intrinsic resistance to the *Cryptococcus* genus [6]. The commercial access of flucytosine in some low-income countries, as well as the heteroresistance found in the use of FLZ, exacerbate this scenario [9,10].

Clioquinol was widely used in the 1950s for the oral treatment of parasitic infections in Japan [11]. Reports of toxicity after exposure to high cumulative doses of the drug occurred almost exclusively in Japanese people and its use was discontinued. Clioquinol has reemerged as a therapeutic agent for diseases beyond parasitic infection, including cancer and Alzheimer’s disease, largely due to its metal-chelating properties [12,13,14]. 8-hydroxyquinoline derivatives, as well as clioquinol, have been extensively investigated in the literature due to their antimicrobial potential. Studies report their activity against mycobacteria [15], bacteria [16,17], viruses [18,19], parasites [20,21] and fungi [22,23,24]. Although antifungal activity has already been reported for CQ and 8HQ against *Cryptococcus* species [25,26,27], the combination of CQ and 8HQ with FLZ/AMB remains unstudied.

The scarcity of options for treating cryptococcosis, and the high cost and lengthy development time of new drugs, make combinations and drug repositioning promising alternatives [28,29,30]. In this study, combinations of the antifungal agents FLZ and AMB with clioquinol (CQ) and 8-hydroxyquinoline (8HQ) were evaluated, chosen for their known antifungal activity and ability to cross the blood–brain barrier. The objective was to associate different mechanisms of action to enhance activity, reduce doses and toxicity, and prevent resistance, aiming at a multi-target treatment for cryptococcosis.

## 2. Materials and Methods

### 2.1. Strains and Growth Medium

For this study, strains H99 (*C. neoformans* var. *grubii*), MYA 4093 (*C. gattii*), B3501 (*C. neoformans* var. *deneoformans*), ATCC32045 (*C. neoformans*), and the clinical isolates LB619 (*C. neoformans* var. *grubii*), LB141 (*C. neoformans*) and LB218 (*C. gattii*) were used. These were found in the Applied Mycology Research Laboratory at the Federal University of Rio Grande do Sul. All clinical isolates were identified using MALDI-ToF (Bruker, Bremen, Germany). Clinical strains and isolates were cultivated prior to testing on Sabouraud Dextrose Agar (SDA) (Kasvi, Pinhais, Brazil) at 35 °C for 48 h. Antimicrobial Sensitivity Test, Checkerboard Combination Assay, time–kill combination curves, and UV/Vis detection of FLZ and CQ/8HQ through designed experiments were performed using RPMI-1640 with 0.03% (*w*/*v*) L-glutamine culture medium (Gibco, Grand Island, NY, USA). RPMI was buffered with 0.165 M MOPS (Neon, Suzano, Brazil), adjusted to pH 7.0, and supplemented with 2% (*w*/*v*) d-glucose (Neon, Suzano, Brazil). The inhibition of melanization test was performed using chemically defined minimal medium (MM) agar (15 mM dextrose (Neon, Suzano, Brazil), 10 mM MgSO_4_ (Synth, Diadêma, Brazil), 29.4 mM KH_2_PO_4_ (Dinâmica, Indaiatuba, Brazil), 13 mM glycine (Dinâmica, Indaiatuba, Brazil), 3 μM thiamine hydrochloride (Êxodo, Sumaré, Brazil), pH 5.5, and 1.5% agar (Sigma-Aldrich, St. Louis, MO, USA). Urease production inhibition tests were performed using commercial microbiological urea broth (yeast extract 0.1 g/L; potassium dihydrogen phosphate 9.1 g/L; disodium hydrogen phosphate 9.5 g/L; urea 20.0 g/L; phenol red 0.01 g/L) (Sigma-Aldrich, St. Louis, MO, USA).

### 2.2. Antifungal Drugs

**Figure 1:**
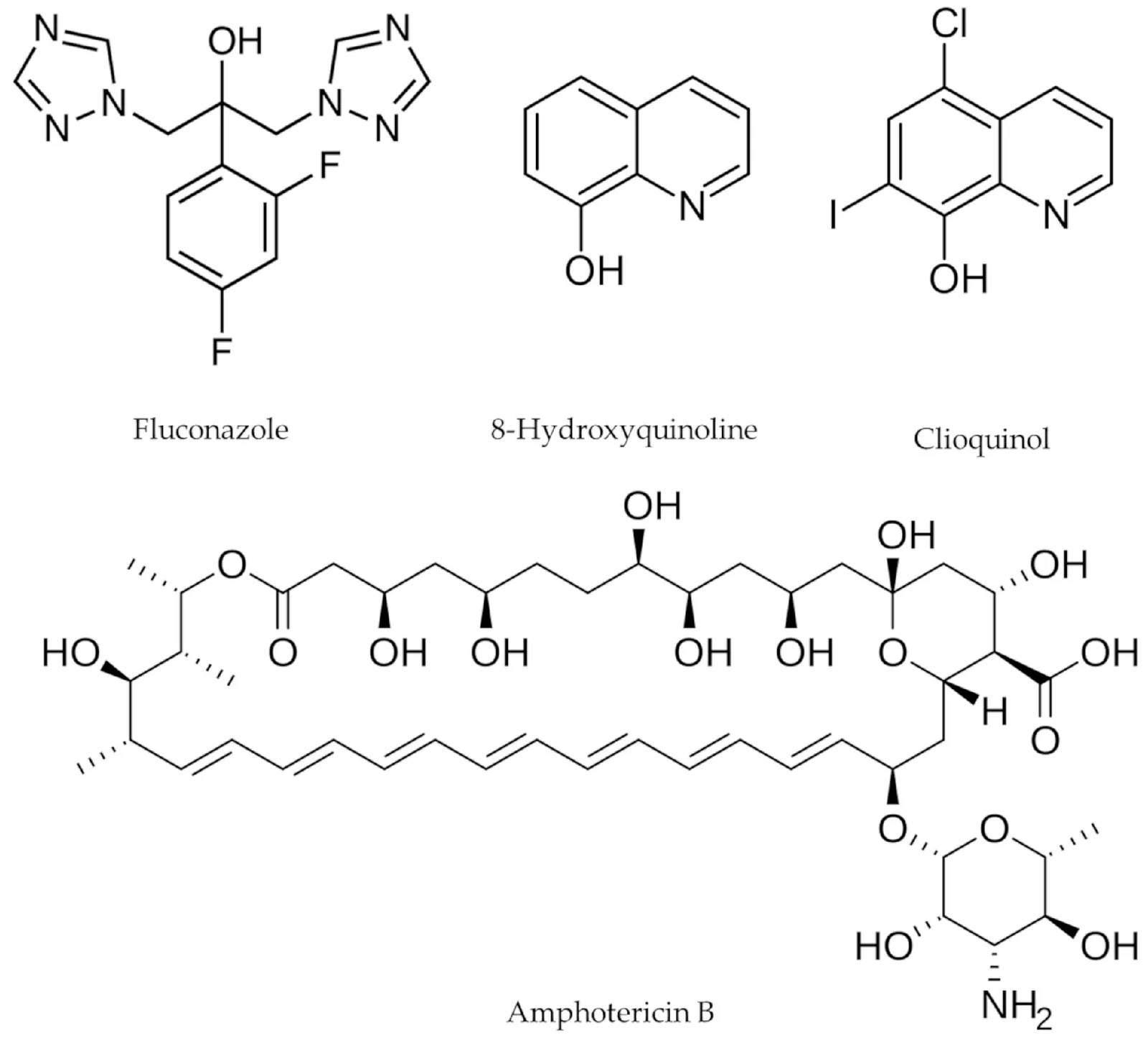
Structure of the molecules used in the study.

Susceptibility and combination tests were performed using the commercial antifungal agents (Figure 1) fluconazole (Pfizer, Itapevi, Brazil) and amphotericin B deoxycholate (Cristália, Itapira, Brazil) (Figure 1). Clioquinol and 8-hydroxyquinoline were purchased from Sigma-Aldrich, USA. Stock solutions were prepared in 100% Dimethyl sulfoxide (DMSO) (Neon, Suzano, Brazil) at concentrations of 2 to 5 mg.mL^−1^ and stored at −20 °C until use. For all assays performed using stock solutions of these antifungals, working solutions were prepared with a final DMSO solvent concentration of below 1% in order to avoid any cytotoxic effect on fungal cells [31].

### 2.3. Antimicrobial Sensitivity Test (AST)

AST was performed using the broth microdilution methodology, following the EUCAST E.DEF 7.3.2 protocol [31]. Seven strains were used: H99, MYA4093, LB218, LB619, LB141, B3501, and ATCC32045. The strains were previously grown on SDA (Kasvi, Pinhais, Brazil) for 48 h at 35 °C. Five colonies were suspended in sterile deionized water and the inoculum adjusted to 3.7 × 10^6^ cells·mL^−1^ using a spectrophotometer (Visible Spectrophotometer, Global Trade Technology, São Paulo, Brazil) at a wavelength of 530 nm. The inoculum solution was prepared in RPMI 1640 medium (Gibco, Grand Island, NY, USA) (1:10) and 100 µL was subsequently added to the already microdiluted drugs, reaching a final cell concentration of 1.7 × 10^5^ CFU/mL in each well of the 96-well polystyrene plate. The molecules were serially diluted for the experiments to concentrations of 4 µg·mL^−1^ for AMB, 32 µg·mL^−1^ for FLZ, 16 µg·mL^−1^ for 8HQ, and 8 µg·mL^−1^ for CQ. ASTs were read 48 h after incubation at 35 °C using a plate reader (Kasuaki, Santa Catarina, Brazil). Absorbances (620 nm) was used to obtain an inhibitory concentration of 50% (IC_50_) and 90% (IC_90_) using the GraphPad Prism 8 software (GraphPad software Inc., La Jolla, CA, USA). Data were normalized, and antifungal concentrations were log-transformed. The inhibition dose–response curves were obtained using the log(inhibitor) vs. normalized response–variable slope model. The IC_50_ determination was obtained to allow for the planning and performance of the combination tests.

### 2.4. Checkerboard Combination Assay

The combination tests were performed according to the checkerboard methodology [32,33] using the broth microdilution technique, as previously mentioned in Section 2.3 [31]. Initially, strains H99 and MYA4093 were tested in FLZ + CQ, FLZ + 8HQ, AMB + CQ, FLZ + AMB, AMB + 8HQ, and CQ + 8HQ combinations. The microdilution of the drugs was performed using working solutions in RPMI 1640 medium (Gibco, Grand Island, NY, USA) that was 2× more concentrated considering the micro-dilution (seven dilutions) of drugs A and B (Figure S1), with the subsequent addition of 100 µL of inoculum (final concentration of 1.7 × 10^5^ CFU/mL). The antifungal concentrations tested were as follows.

Strain H99: 2 to 0.03 μg/mL for CQxAMB; 4 to 0.06 μg/mL^−1^ for CQx8HQ; 2 to 0.03 μg/mL^−1^ and 4 to 0.06 μg/mL^−1^, respectively, for AMBx8HQ; 8 to 0.125 μg/mL^−1^ and 4 to 0.06 μg/mL^−1^, respectively, for FLZxCQ; 16 to 0.25 μg/mL^−1^ and 4 to 0.06 μg/mL^−1^, respectively, for FLZx8HQ; 8 to 0.125 μg/mL^−1^ and 2 to 0.03 μg/mL^−1^, respectively, for FLZxAMB.

MYA4093 strain: 2 to 0.03 μg/mL^−1^ for CQxAMB; 4 to 0.06 μg/mL^−1^ for CQx8HQ; 2 to 0.03 μg/mL^−1^ and 4 to 0.06 μg/mL^−1^, respectively, for AMBx8HQ; 8 to 0.125 μg/mL^−1^ and 4 to 0.06 μg/mL^−1^, respectively, for FLZxCQ; 8 to 0.125 μg/mL^−1^ and 4 to 0.06 μg/mL^−1^, respectively, for FLZx8HQ; 8 to 0.125 μg/mL^−1^ and 2 to 0.03 μg/mL^−1^, respectively, for FLZxAMB.

The concentrations tested for each molecule were adjusted according to the susceptibility profile of the strains obtained by AST, described in Section 2.3. A flat-bottom 96-well plate was used for the assay, according to the layout in Figure S1. Drug A was added in column 8 and microdiluted to column 2. Then drug B was microdiluted from row H to row B. Column 1 shows the control for drug B alone, and row A shows the control for the drug A alone. Tests were performed in triplicate. After 48 h of incubation at 35 °C, the plates were read using a plate reader (Kasuaki, Santa Catarina, Brazil) at 620 nm. The results were normalized and analyzed in the Combenefit (2.021 version, 2016) software using the Bliss-independent model to study the combinations [34]. After executing the combinations for H99, and MYA4093, other strains were tested to determine the best combination results. The strains used were LB218 (*C. gattii*), LB619 (*C. neoformans* var. *grubii*), LB141 (*C. neoformans*), B3501 (*C. neoformans* var. *deneoformans*), and ATCC32045 (*C. neoformans*), keeping the same microdilution scheme shown in Figure S1. The tests were performed in triplicate.

### 2.5. Modified Time–Kill Combination Curves

This test was carried out according to the technique previously mentioned in Section 2.3, Antimicrobial Sensitivity Test, and 2.4, Checkerboard Combination Assay. This assay was based on the study by Prasad et al. [35] and adapted for use with *Cryptococcus* strains, using RPMI 1640 (Gibco, Grand Island, NY, USA) culture medium, incubation at 35 °C and fungal inoculum of 1.7 × 10^5^ CFU/mL. The combination of FLZ and CQ was established with H99 and MYA4093 strains. The plates were read in a microplate reader at 620 nm (Kasuaki, Santa Catarina, Brazil) at 0, 16, 20, 24, 39, and 44 h. To assess cell viability, paired plates with the vital dye resazurin (0.01%) were used, read at 620 nm in a microplate analyzer at the same time intervals.

This adaptation to the conventional time–kill assay by counting Colony-Forming Units (CFU) was used to reduce the high consumption of materials, agar plates, and time. Although this technique has the limitation of not being able to determine the number of dead cells, we can quantify the metabolism of viable cells instantaneously, while CFU estimates depend on cell regrowth after the incubation time. Other limitations, such as the difficulty in defining the ideal dilution range and cumulative errors due to repeated pipetting, interfere with CFU. It should also be noted that viable cells capable of metabolizing resazurin may not form colonies after exposure to the drug, underestimating the viability that is closest to the real value. Furthermore, residual drug present in the dilutions can inhibit colony formation, compromising the accuracy of the count, as well as making the number of cells that originated in each CFU uncertain and unknown. Therefore, the time–kill technique using resazurin provides us with more reliable and practical data.

All the tests were performed in quadruplicate. Data were normalized, and antifungal concentrations were log-transformed. The dose–response curves were obtained using the log (inhibitor) vs. normalized response–variable slope model.

### 2.6. Inhibition of Melanization

The inhibition of melanin production by the derivatives was achieved using the protocol described by Baker et al. [36] with modifications. *C. neoformans* (H99) and *C. gattii* (MYA4093) cells were harvested during the exponential growth phase and resuspended at 3.7 × 10^6^ CFU/mL sterile deionized water. Then, 24-well polystyrene plates were filled with 2 mL per well of chemically defined minimum medium agar (MM) (15 mM dextrose, 10 mM MgSO_4_, 29.4 mM KH2PO4, 13 mM glycine, 3 μM thiamine hydrochloride, pH 5.5, and 1.5% agar) with 1.0 mM 3,4-dihydroxy-L-phenylalanine (L-DOPA, Sigma-Aldrich). The addition of thiamine, L-DOPA, and different concentrations of FLZ, CQ, and 8HQ to the MM agar was performed before the solidification process. This process was carried out to avoid the degradation of these molecules at the high temperatures reached during the melting of the agar. After the MM agar solidified, a 10 µL aliquot of the *C. neoformans* and *C. gattii* inoculum suspension was deposited in each well in 24-well plates containing MM agar with concentrations of 2, 4, and 8 µg·mL^−1^ for FLZ, 0.25, 0.5, and 1 µg·mL^−1^ for CQ, and 0.12/0.5, 0.25/1, and 0.5/2 µg·mL^−1^ for FLZ/CQ, in the presence and absence of 1 mM L-DOPA (Sigma-Aldrich, USA). Plates were incubated at 35 °C, and read after 7 days. The experiments were performed in triplicate.

### 2.7. Inhibition of Urease Production

The evaluation of the inhibition of urease enzyme production by *C. neoformans* (H99) and *C. gattii* (MYA 4093) was performed by microdilution assay using urea broth (yeast extract 0.1; potassium dihydrogen phosphate 9.1; di-sodium hydrogen phosphate 9.5; urea 20.0; phenol red 0.01, Sigma-Aldrich, USA) [37]. The inoculum was adjusted to a final concentration of approximately 1 × 10^5^ CFU/mL. CQ, FLZ, and FLZ + CQ were tested at 8 to 0.06 µg·mL^−1^, 32 to 0.25 µg·mL^−1^, and 2/8 to 0.03/0.06 µg·mL^−1^, respectively. The results were read at 620 and 492 nm using a microplate reader (Kasuaki, Santa Catarina, Brazil) after six days of incubation at 35 °C.

The optical densities were plotted on Microsoft Excel (Microsoft, Redmond, WA, USA). The growth control (medium and inoculum) and medium control (medium only) were obtained as means, coefficient of variation, and upper and lower confidence intervals (95%). Through the confidence intervals obtained for the controls (statistically different), a matrix was assembled by a function in which for samples that present ODs greater than or equal to the lower confidence interval of the growth control, urea conversion occurs. If lower, there was no urea hydrolysis (no urease activity). Afterward, the results were investigated for the inhibition of urease activity in the samples that showed conversion. Samples with ODs lower than, or equal to, the upper confidence interval of the medium control were considered to show inhibition. If higher, there was no inhibition. The experiments were performed in triplicate.

### 2.8. UV/Vis Detection of FLZ and CQ/8HQ by Experimental Design

The activity of combinations between FLZ with CQ and FLZ with 8HQ was analyzed regarding their action on fungal cells after cultivation in RPMI 1640 medium through the designed experiments. *C. neoformans* and *C. gattii* were cultivated at 35 °C for 48 h in macro-dilution (8 mL), containing combinations of FLZ + CQ/ and FLZ + 8HQ at concentrations of 2 and 8 µg·mL^−1^ to 0.03 and 0.125 µg·mL^−1^, respectively, and fungal inoculum of 1 × 10^5^ CFU/mL.

After 48 h of incubation at 35 °C, the samples, in their respective concentrations, were centrifuged at 3000 rpm for 10 min. The supernatant was transferred to a new sterile tube (4 mL), and the samples’ supernatants were read on a UV-1800 spectrophotometer (Shimadzu, Kyoto, Japan) in scan mode from 200 to 800 nm, with intervals of 1 nm.

The combination drugs (FLZ + CQ/8HQ) were also analyzed in RPMI 1640 medium without fungal cells. The drugs added to the culture medium with fungal cells according to the design are shown in the Table 1 below.

The obtained spectra were imported into the GraphPad Prism software and analyzed according to their profile. This assay was based on the likely mechanism of the chelating action of CQ and 8HQ, as reported in the literature [38]. The experiments were performed in triplicate.

### 2.9. Hen’s Egg Test–Chorioallantoic Membrane (HET-CAM)

White fertile eggs from Lohmann (Lohmann selected Leghorn, LSL) were incubated until ten days at 38 to 39 °C, at a humidity between 55 and 60%. On the 10th day, the eggshell was carefully removed around the airspace with a rotary tool (Dremel, Mount Prospect, IL, USA). Subsequently, 300 µL of each solution was added to each egg. Phosphate-buffered saline was used as the negative control and 0.1 M of NaOH as the positive control. FLZ was tested at 16 µg·mL^−1^, CQ at 2 µg·mL^−1^, and the combination at 0.125 + 0.25 µg·mL^−1^, respectively. The irritant effect was observed 30 s, 2, and 5 min after the application of each substance. The irritation score (*IS*) was calculated according to the equation below. A scale from 0 to 4.9 denoted nonirritant (or practically no irritation), and 5.0 to 21 denoted irritant (moderate/severe or extreme irritation) [39].

$$IS=\left(\left(\frac{\left(301-Hemorrhage\;Time\right)}{300}\right)\times 5\right)+\left(\left(\frac{\left(301-Lysis\;Time\right)}{300}\right)\times 7\right)+\left(\left(\frac{\left(301-Coagulation\;Time\right)}{300}\right)\times 9\right)$$

### 2.10. Cytotoxicity Assay

The 3T3 cell line was routinely cultured in 75 cm^2^ flasks using DMEM supplemented with 10% heat-inactivated fetal bovine serum (FBS, Sigma, St. Louis, MO, USA), 100 U mL^−1^ penicillin (Gibco, Paisley, UK), and 100 mg mL^−1^ streptomycin (Gibco, Paisley, UK). The cells were maintained at 37 °C in a humidified 5% CO_2_–95% air atmosphere. The cells were fed every 2 or 3 days and subcultured when reaching 70–80% confluence. The cytotoxicity was evaluated through the previously described MTT reduction assays [40]. The cells were seeded at a density of 100,000 cells per well in (96-well plates). A total of 1% of triton X-100 (Sigma-Aldrich, St. Louis, MO, USA) was used as a positive control. Negative control cells were incubated in a culture medium. Cytotoxicity was evaluated by incubating the cells with different concentrations of clioquinol, amphotericin B, fluconazole, and a combination of clioquinol and fluconazole for 24 h at 37 °C. Solutions for the molecules were made in DMSO. The solvent control used was 0.1% of DMSO [40].

### 2.11. Acute Toxicity Tests on Tenebrio Molitor Larvae

*Tenebrio molitor* larvae were used to assess acute systemic toxicity. The larvae were anesthetized through cooling (2 °C) for 2 min. Afterward, 50 μL of the solutions (FLZ, CQ, FLZ + CQ, and saline solution) were added to the hemocoel with a micro-syringe and inserted in the second or third visible sternite at the ventral portion. A total of 0.9% saline solution was used as a negative control. FLZ and CQ drugs were tested at concentrations of 16 and 2 µg·mL^−1^, respectively, and their combination was tested at concentrations of 0.125 and 0.25 µg·mL^−1^, respectively. Larvae were incubated in Petri dishes at 37 °C and fed a rearing diet. The larvae were counted at four-hour intervals over two days [41,42]. Tests were conducted in triplicate on groups of six larvae. Statistical analysis was performed using the Chi-square test, with *p* < 0.05 considered significant.

## 3. Results

### 3.1. Inhibitory Concentration of Antifungal Drugs

The antifungal activity found in our study for 8HQ and CQ (Table 2) was satisfactory, reaching inhibitory concentrations better than, or as potent as, the drugs tested (FLZ and AMB).

### 3.2. Drug Interaction Response by Checkerboard Assay

**Figure 2:**
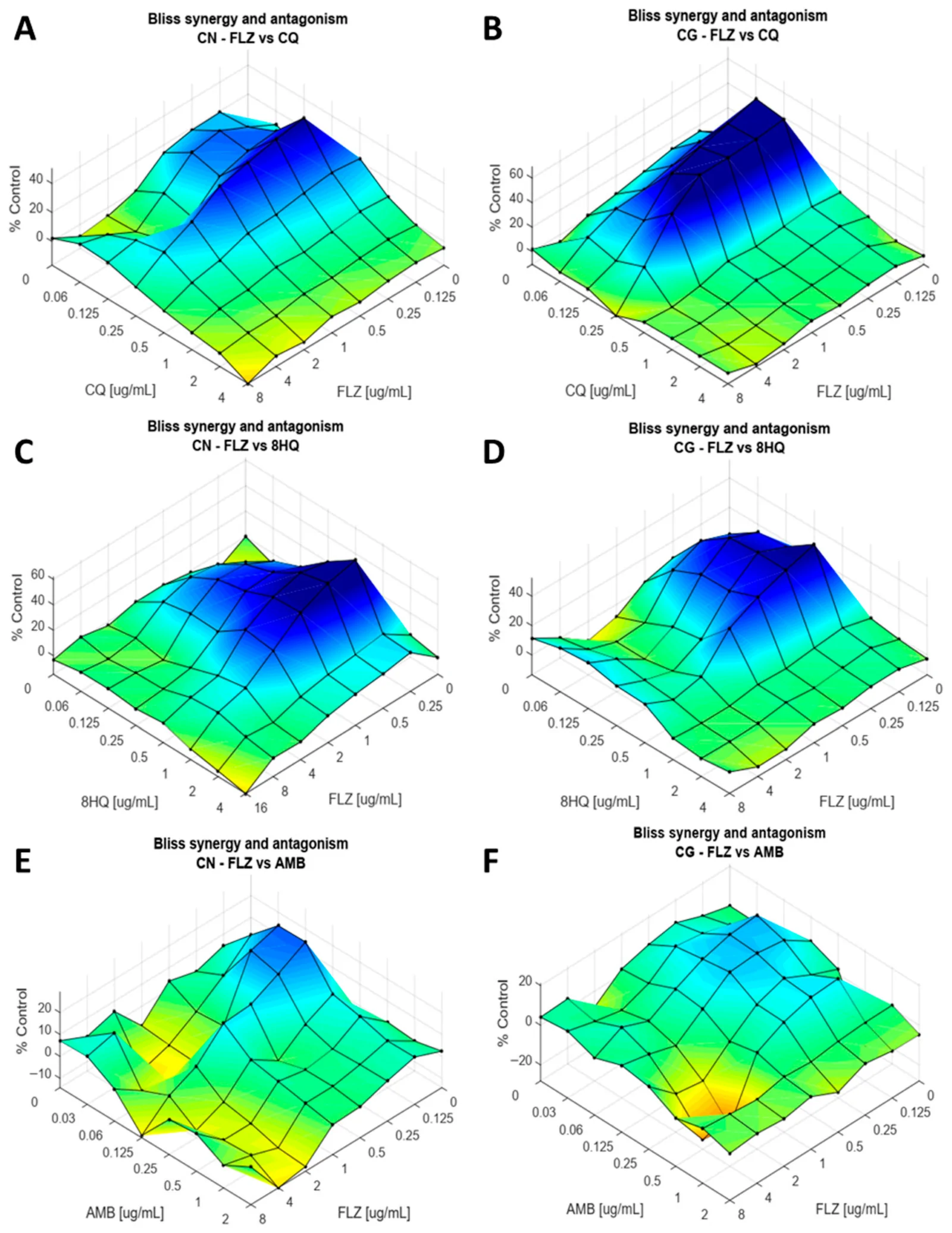
Tested combinations for strains H99 (*C. neoformans* var. *grubii*) and MYA4093 (*C. gattii*): (**A**) combination of FLZ + CQ for H99; (**B**) combination of FLZ + CQ for MYA4093; (**C**) combination of FLZ + 8HQ for H99; (**D**) combination of FLZ + 8HQ for MYA4093; (**E**) combination of FLZ + AMB for H99; (**F**) combination of FLZ + AMB for MYA4093; CN: H99; CG: MYA4093. Blue: the stronger the shade of blue, the greater the synergistic effect found. Green: the green shade indicates an additive effect. Yellow-red: yellow shades tending towards orange/red indicate a greater antagonistic effect.

After obtaining an inhibitory concentration of 50%, combinations of FLZ, AMB, CQ, and 8HQ were tested. The combination FLZ and CQ for the H99 strain showed a region of strong synergism at a concentration of 0.25 µg·mL^−1^ of CQ (Figure 2A). FLZ, on the other hand, plays a dose-dependent role in synergism, where the region of synergism extends to be between 1 and 0.125 µg·mL^−1^ of FLZ. The intensity of synergism increases as FLZ concentrations decrease. The highest synergism is obtained with 0.125 µg·mL^−1^ of FLZ and 0.25 µg·mL^−1^ of CQ (score 50 ± 2, *p* < 0.01, Figure S2). A region of slight antagonism can be seen at the highest concentrations (2–8 µg·mL^−1^ of FLZ and 4 µg·mL^−1^ of CQ).

The MYA4093 strain shows a substantial region of synergism between the concentrations of 0.125 to 0.25 µg·mL^−1^ for CQ, and an increasing synergism response at the lowest concentrations of FLZ, 2 to 0.125 µg·mL^−1^ (Figure 2B). The best synergism occurs between 0.125 µg·mL^−1^ of FLZ and 0.25 µg·mL^−1^ of CQ (score 66 ± 1, *p* < 0.001, Figure S3).

The combination of FLZ + 8HQ for H99 (Figure 2C) also exhibits a region of high synergism between concentrations of 0.125 to 1 µg·mL^−1^ of 8HQ and 0.25 to 2 µg·mL^−1^ of FLZ. The best score (62 ± 1, *p* < 0.001, Figure S4) can be observed at concentrations of 0.25 and 1 µg·mL^−1^ of FLZ and 8HQ, respectively. For MYA4093, the region of synergism located between 0.125 and 0.5 µg·mL^−1^ of 8HQ and 0.125–1 µg·mL^−1^ of FLZ. The most significant synergism occurred between concentrations of 0.125 and 0.5 µg·mL^−1^ of FLZ and 8HQ (Figure 2D), respectively (score 52 ± 4, *p* < 0.05, Figure S5).

In the combination of FLZ + AMB, the synergism found for H99 is moderate to weak; the region is present between the concentrations of 0.06–0.125 µg·mL^−1^ of AMB and 0.125–1 µg·mL^−1^ of FLZ (Figure 2E). The best synergism was observed between 0.06 and 0.125 AMB with 0.125 µg·mL^−1^ FLZ (score 29 ± 4, *p* < 0.05, Figure S6). A region of mild antagonism can be seen at 0.5–2 µg·mL^−1^ of AMB and 2–4 µg·mL^−1^ of FLZ. The MYA4093 strain also has a weak region of synergism between 0.125 µg·mL^−1^ and 0.06 µg·mL^−1^ of FLZ and AMB, respectively (Figure 2F). A point of moderate antagonism can be observed between 2 and 0.5 µg·mL^−1^ of FLZ and AMB, respectively.

**Figure 3:**
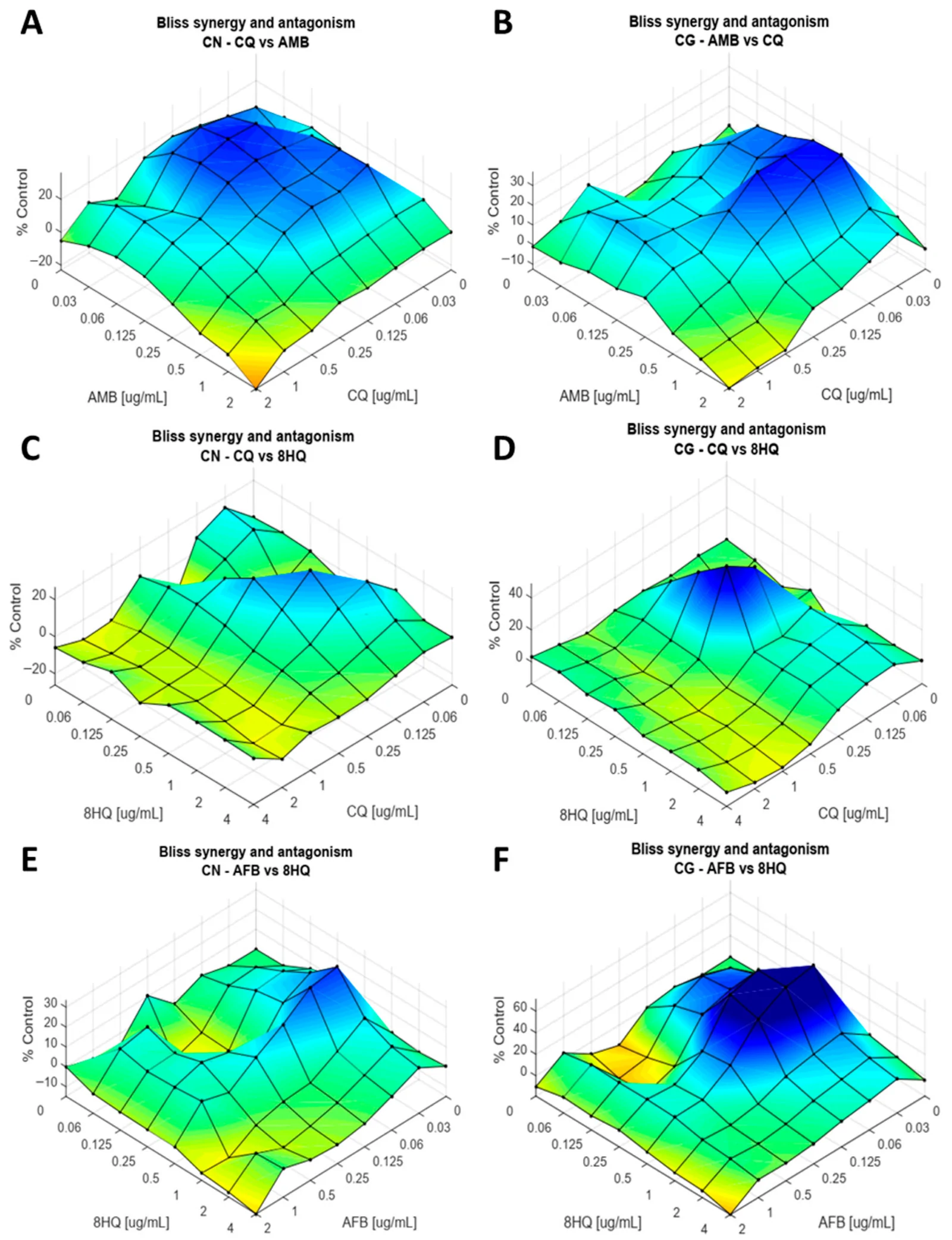
Performed combinations for H99 (*C. neoformans* var. *grubii*) and MYA4093 (*C. gattii*) strains: (**A**) combination of CQ + AMB for H99; (**B**) combination of CQ + AMB for MYA4093; (**C**) combination of CQ + 8HQ for H99; (**D**) combination of CQ + 8HQ for MYA4093; (**E**) combination of AMB + 8HQ for H99; (**F**) combination of AMB + 8HQ for MYA4093; CN: H99; CG: MYA4093. Blue: the stronger the shade of blue, the greater the synergistic effect found. Green: the green shade indicates an additive effect. Yellow-red: yellow shades tending towards orange/red indicate a greater antagonistic effect.

The combination AMB vs. CQ for H99 shows a region of moderate synergism at the concentrations of 0.03–0.25 µg·mL^−1^ of CQ and 0.03–0.5 µg·mL^−1^ of AMB (Figure 3A), and the best concentration was 0.03 and 0.5 µg·mL^−1^ of CQ and AMB, respectively (score 30 ± 1, *p* < 0.01, Figure S8).

A region of mild to moderate antagonism is present at concentrations of 1–2 µg·mL^−1^ of AMB and 0.5–2 µg·mL^−1^ of CQ. For strain MYA4093, as well as H99, a moderate region of synergism is observed (Figure 3B). The concentrations of this region are between 0.03–0.25 µg·mL^−1^ of CQ and 0.06–0.5 µg·mL^−1^ of AMB, with the best concentrations being 0.03 and 0.5 µg·mL^−1^ of CQ and AMB (score 37 ± 4, *p* < 0.05, Figure S9). It is also possible to visualize a small region of antagonism between 0.5–1 µg·mL^−1^ of CQ and 2 µg·mL^−1^ of AMB.

As for the combination between CQ + 8HQ for H99, a small region of low synergism can be seen (Figure 3C), with the best combination point located at concentrations of 0.125 and 0.5 µg·mL^−1^ of CQ and 8HQ, respectively (score 27 ± 2, *p* < 0.05, Figure S10). A mild region of antagonism between concentrations of 1–2 and 0.5–2 µg·mL^−1^ of CQ and 8HQ are present. Unlike H99, MYA4093 has a strong synergism point at concentrations of 0.25 µg·mL^−1^ (Figure 3D) for CQ + 8HQ (score 49 ± 2, *p* < 0.01, Figure S11).

When we evaluate the combination between AMB + 8HQ, we observe similar behavior. A region of slight synergism can be observed at concentrations between 0.03–0.25 µg·mL^−1^ and 0.5–1 µg·mL^−1^ for AMB vs. 8HQ (Figure 3E), respectively, in H99. The best synergism point occurs at 0.03 and 1 µg·mL^−1^ of AMB and 8HQ, respectively (score 27 ± 3, *p* < 0.05, Figure S12). A slight antagonism is present in the region between 0.5–1 µg·mL^−1^ of AMB and 2–4 µg·mL^−1^ of 8HQ. The MYA4093 strain has a region of strong synergism between 0.03–0.125 µg·mL^−1^ of AMB and 0.25–0.5 µg·mL^−1^ of 8HQ (Figure 3F). The best point of synergism is at concentrations of 0.03 µg·mL^−1^ of AMB and 0.5 µg·mL^−1^ of 8HQ (Scores 71 ± 3, *p* < 0.01, Figure S13), and a point of moderate antagonism is present in the larger concentrations, 2 and 4 µg·mL^−1^, of AMB and 8HQ.

Based on the synergism results obtained for strains H99 and MYA4093, other strains and clinical isolates were tested (Figures S14–S18) for the combination of FLZ + CQ. As expected, it was observed that this combination again presented high regions of synergism, with the best combination points located mainly between 0.06 and 0.5 µg·mL^−1^ of CQ and 0.06–0.5 µg·mL^−1^ of FLZ, showing the best scores between 36 ± 3 and 69 ± 1. It is important to emphasize that the LB218 (*C. gattii*) isolate, which has reduced susceptibility to FLZ, showed a significant region of synergism.

### 3.3. Evaluation of Modified Time–Kill Combination Curves

**Figure 4:**
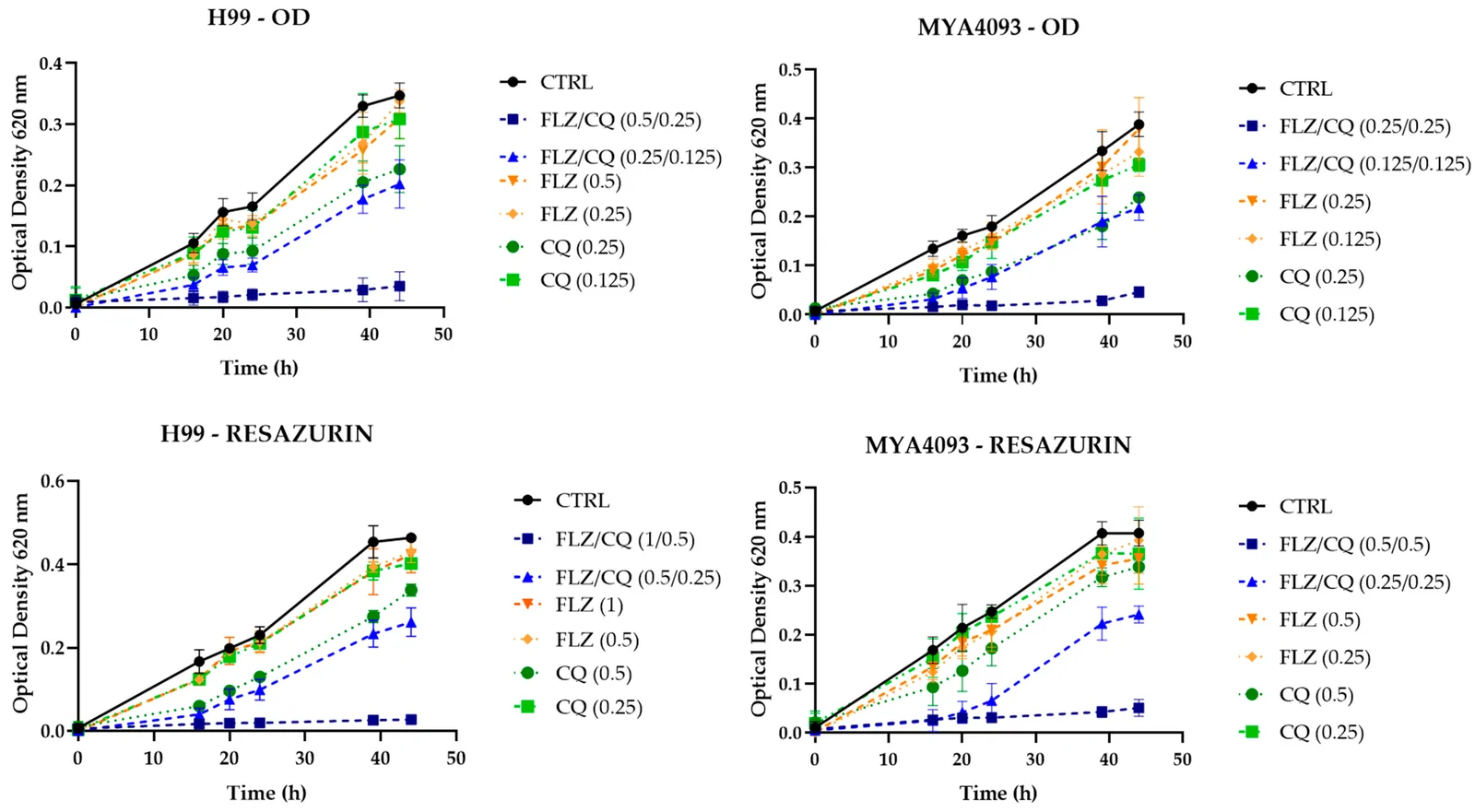
Modified time–kill combination curves of *C. neoformans* and *C. gattii* for FLZ and CQ drugs, alone and in combination, in the presence and absence of the vital dye resazurin.

After performing the association tests, the combination between FLZ + CQ showed the best results and was used to be evaluated according to the action kinetics through time–kill curves for *C. neoformans* and *C. gattii* (Figure 4). The results show that, in general, the combinations were more effective when compared to the drugs alone for all times evaluated for *C. neoformans* (H99). The combination of 0.5 and 0.25 µg·mL^−1^ of FLZ + CQ resulted in 89% inhibition of fungal growth in 44 h, with FLZ alone at a concentration of 0.5 µg·mL^−1^ inhibiting only 11% and CQ 35% with 0.25 µg·mL^−1^, inhibitions 8 and 2.5 times lower than the combination.

However, the presence of the vital dye resazurin showed that although 0.5 and 0.25 µg·mL^−1^ of FLZ + CQ, respectively, caused a significant reduction in fungal growth, the existence of viable cells with an inhibition of only 44% and at twice the concentrations (1 and 0.5 µg·mL^−1^) were necessary to inhibit 94% of the fungal growth. In the presence of the vital dye, FLZ inhibited only 8% of fungal growth and CQ 27%, inhibitions 11.7 and 3.5 times, respectively, lower than their association. This difference in inhibition and concentrations in the presence and absence of vital dye demonstrates the fungistatic pattern of both drugs.

For *C. gattii* (MYA4093), the same behavior presented by *C. neoformans* was observed, although with different concentrations (Figure 4). The combination of FLZ + CQ, in the concentration of 0.25 µg·mL^−1^ caused 88% inhibition of fungal growth in 44 h. Alone, FLZ and CQ inhibited only 28 and 38% of fungal growth. When we analyzed the time–kill curves in the presence of the vital dye, again, the combination containing 0.25 µg·mL^−1^ of both drugs inhibited 37% of the growth, with 0.5 µg·mL^−1^ of both resulting in an inhibition of 87%, while only 13% and 16% reductions in viable cells were observed for the drugs alone.

### 3.4. Melanin Inhibition Assay

**Figure 5:**
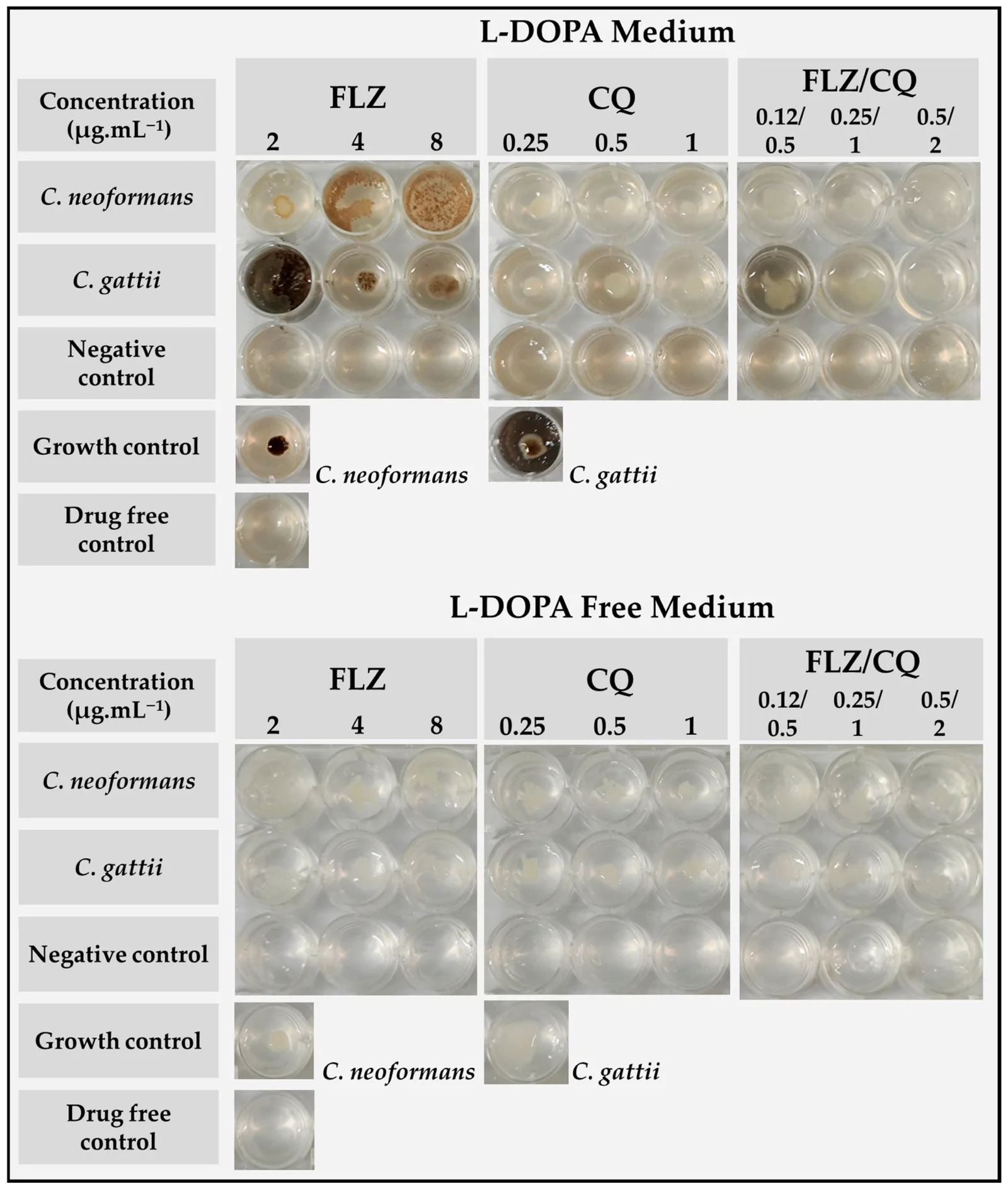
Evaluation of inhibition of melanin production by *C. neoformans* (H99) and *C. gattii* (MYA 4093) in a medium containing 1 mM of L-DOPA or not in the presence of different concentrations of FLZ and CQ drugs, alone and in combination.

The results of the melanin production demonstrate that FLZ alone reduced melanin production for *C. neoformans* (Figure 5) and *C. gattii* in a concentration-dependent manner. In contrast, CQ showed total inhibition of melanin production at all concentrations tested for both strains. The inhibition of melanin production was maintained when the drugs were combined for both strains.

### 3.5. Results for Urease Inhibition

In addition to melanin production, urease activity is an important virulence factor to be studied. In this experiment, it was possible to observe that, for *C. neoformans* (H99), there was no inhibition of urease production (Table 3) by the cells at the tested concentrations. For *C. gattii* (MYA 4093), only two FLZ replicates showed an inhibition of urease activity (Table S1). However, the MYA 4093 strain showed a low production of urease. This fact may have interfered with the analysis.

### 3.6. Quantification of FLZ and CQ/8HQ in RPMI Medium After Incubation

**Figure 6:**
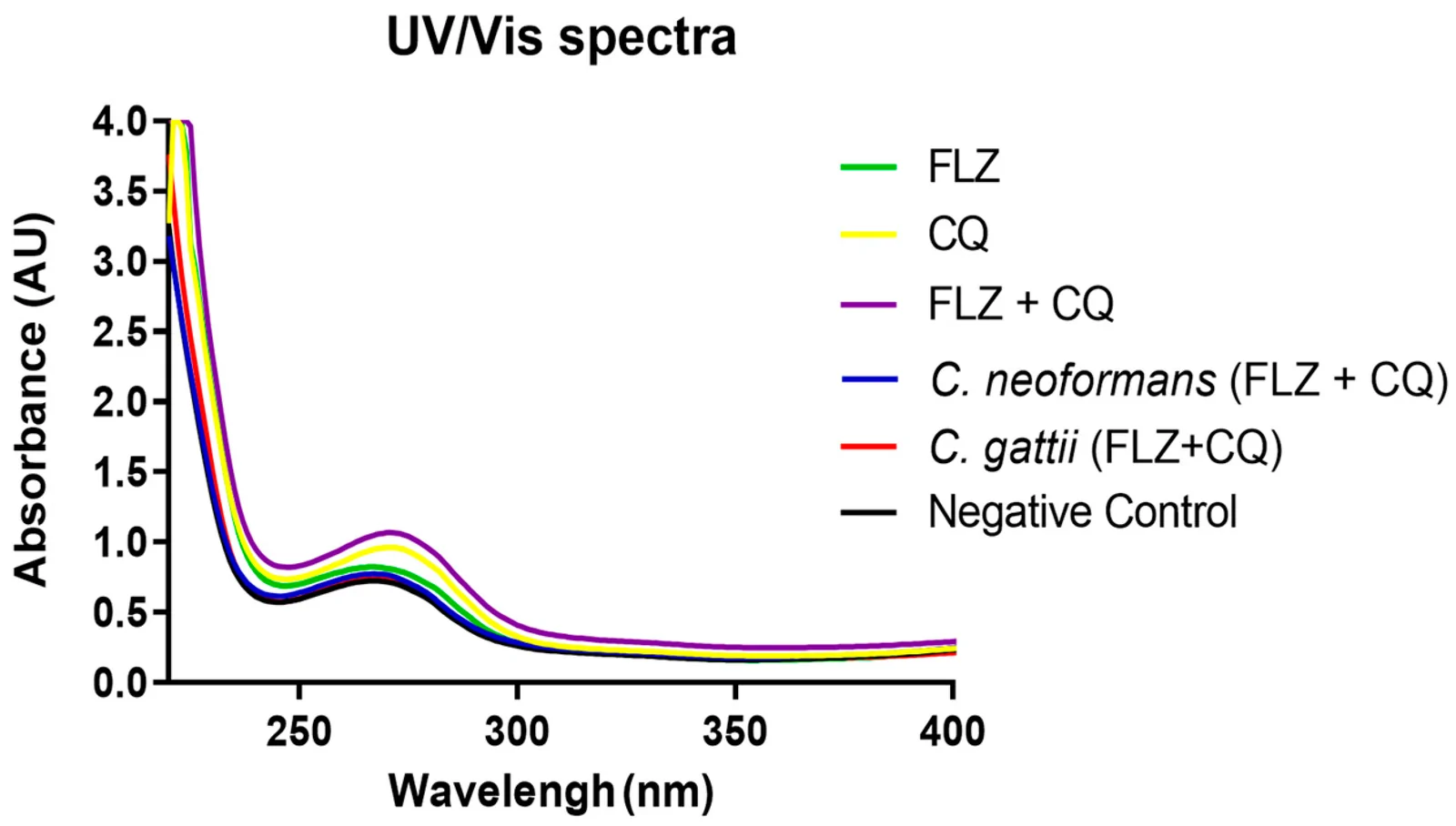
UV/Vis spectra (220–400 nm) of FLZ, CQ, and FLZ + CQ drugs added to the RPMI 1640 culture medium, and samples of *C. neoformans* and *C. gattii* after 48 h of cultivation in the RPMI 1640 medium with drug combination. The tested concentrations of FLZ and CQ in culture with *C. neoformans* and *C. gattii* were 2 and 8 µg·mL^−1^, respectively; CQ in 4 µg·mL^−1^ culture medium; FLZ + CQ in 1.6 and 7.6 µg·mL^−1^ culture medium, respectively; FLZ in 16 µg·mL^−1^ culture medium; negative control: RPMI 1640.

The spectra obtained from the readings of RPMI samples containing the combinations of FLZ + CQ/8HQ after being cultivated in the presence of *C. neoformans* and *C. gattii* cells show that the antifungals in the macro-dilution were not detected (Figure 6 and Figure S19) when compared to antifungals added to the medium, regardless of concentration. These findings may reinforce the entry or binding of drugs to fungal cells in the pellets observed after the sample centrifugation process.

### 3.7. Irritability Test (HET-CAM)

**Figure 7:**
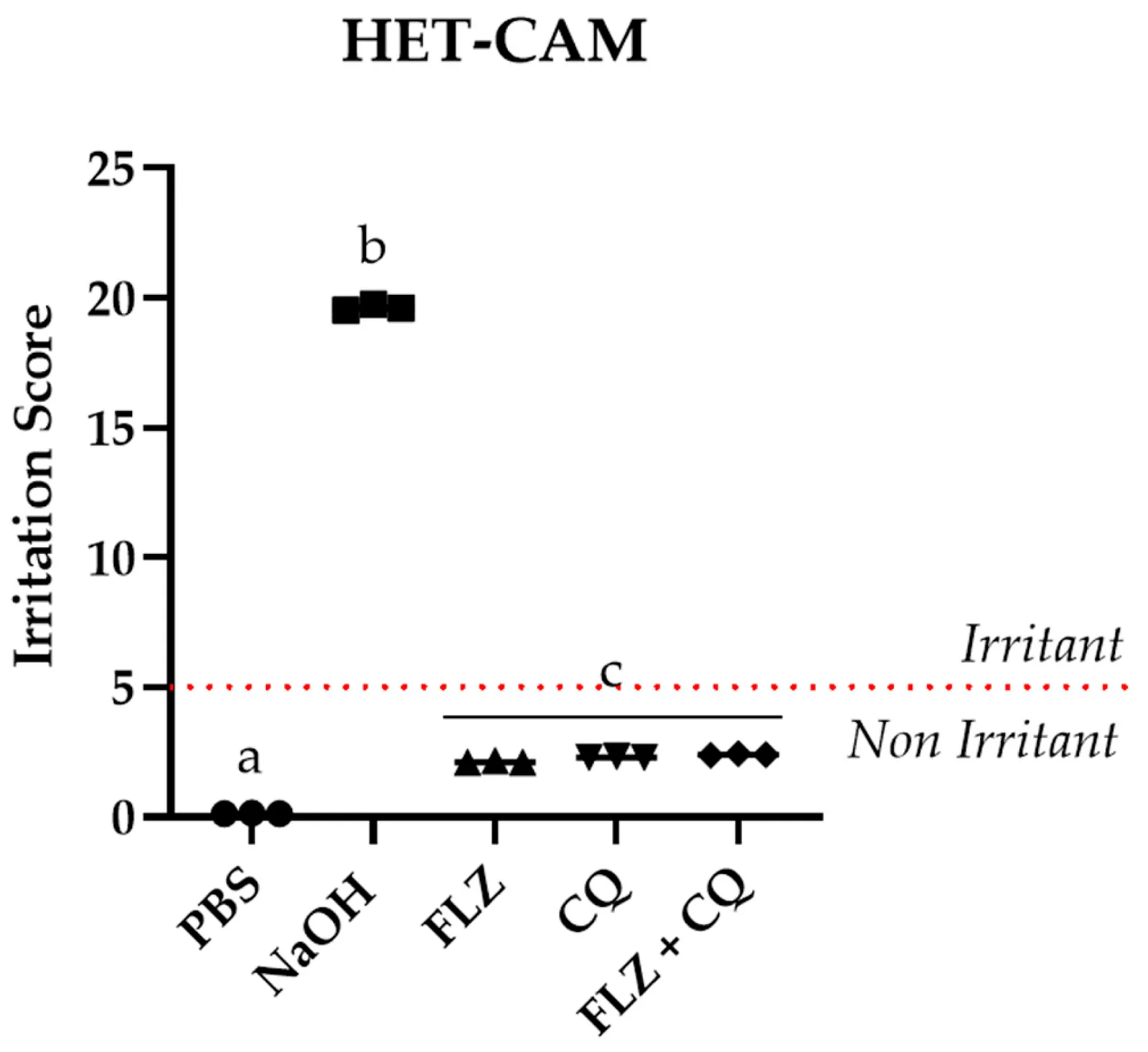
Irritability test performed for FLZ at a concentration of 16 µg·mL^−1^, CQ 2 µg·mL^−1^ and its combination 0.125 + 0.25 µg·mL^−1^. Positive control: 1 mM NaOH. Negative control: PBS. The letters indicate statistical difference (*p* < 0.05). The red dashed line indicates the irritation score.

Analyzing the HET-CAM results, it was possible to verify that FLZ and CQ alone, as well as in combination, did not present an irritating action on the chorioallantoic membrane of embryonated eggs compared to the positive and negative controls. FLZ, CQ, and their combination did not show a statistically significant difference between them, but showed a statistically significant difference in comparison with the controls PBS and NaOH (Figure 7).

### 3.8. Toxicity Results in 3T3 Cells

**Figure 8:**
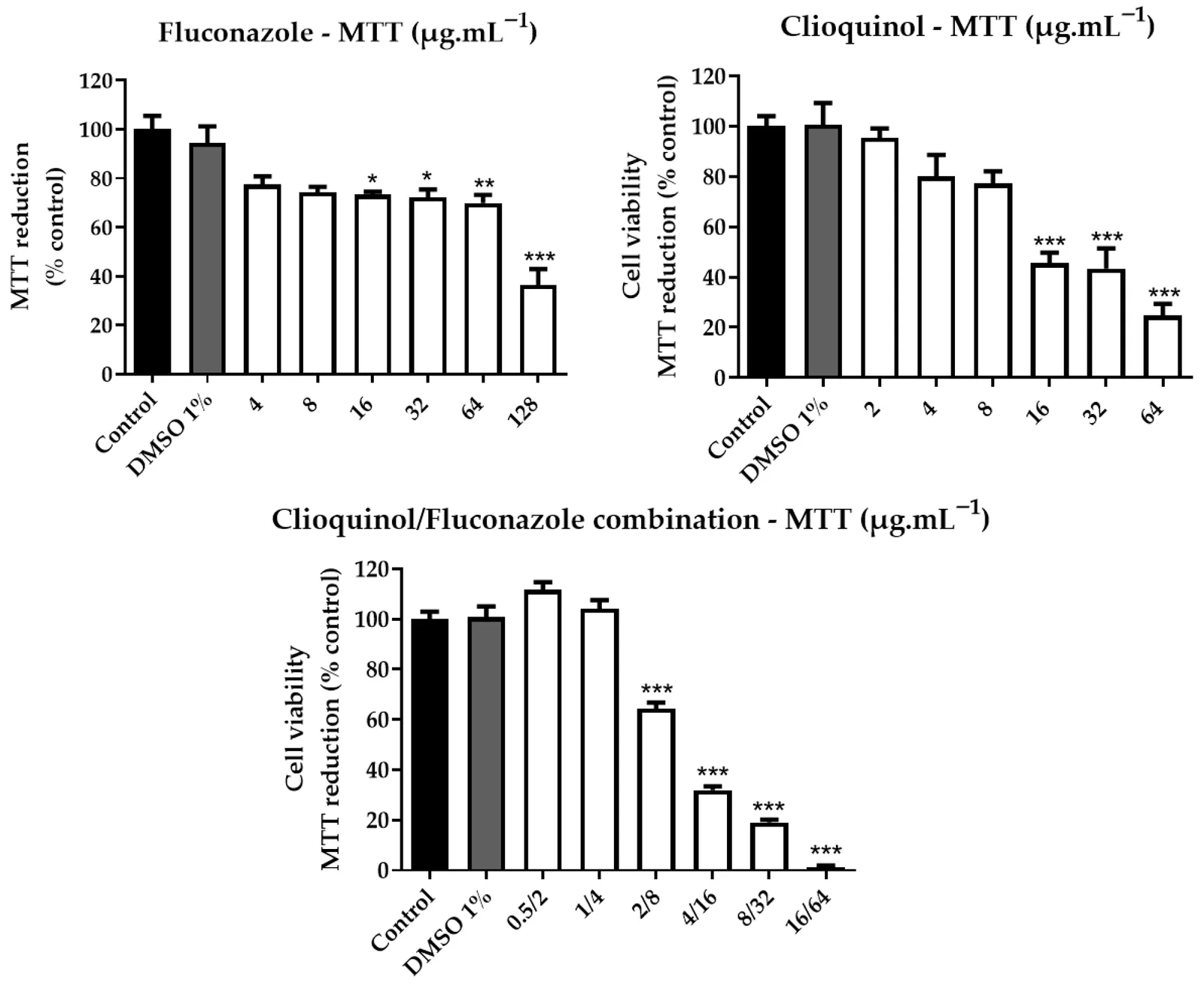
Cytotoxicity assay with mouse embryonic fibroblast cells (3T3 cells) exposed to different concentrations of FLZ, CQ, and FLZ + CQ during 24 h. Solvent control: 0.1% of DMSO. Control: culture medium. The asterisks indicate statistical significance. One asterisk: *p* < 0.05; Two asterisks: *p* < 0.01; Three asterisks: *p* < 0.001.

Cytotoxicity tests on mouse embryonic fibroblast cells (3T3 cells) show that combinations at concentrations of 1 µg·mL^−1^ of CQ and 4 µg·mL^−1^ of FLZ did not reduce cell viability (Figure 8). These concentrations are 4 and 32 times higher than the region of best synergism found for *C. neoformans* and *C. gattii* (0.125 µg·mL^−1^ of CQ and 0.25 µg·mL^−1^ of FLZ).

### 3.9. Acute Toxicity Tests on Tenebrio Molitor

**Figure 9:**
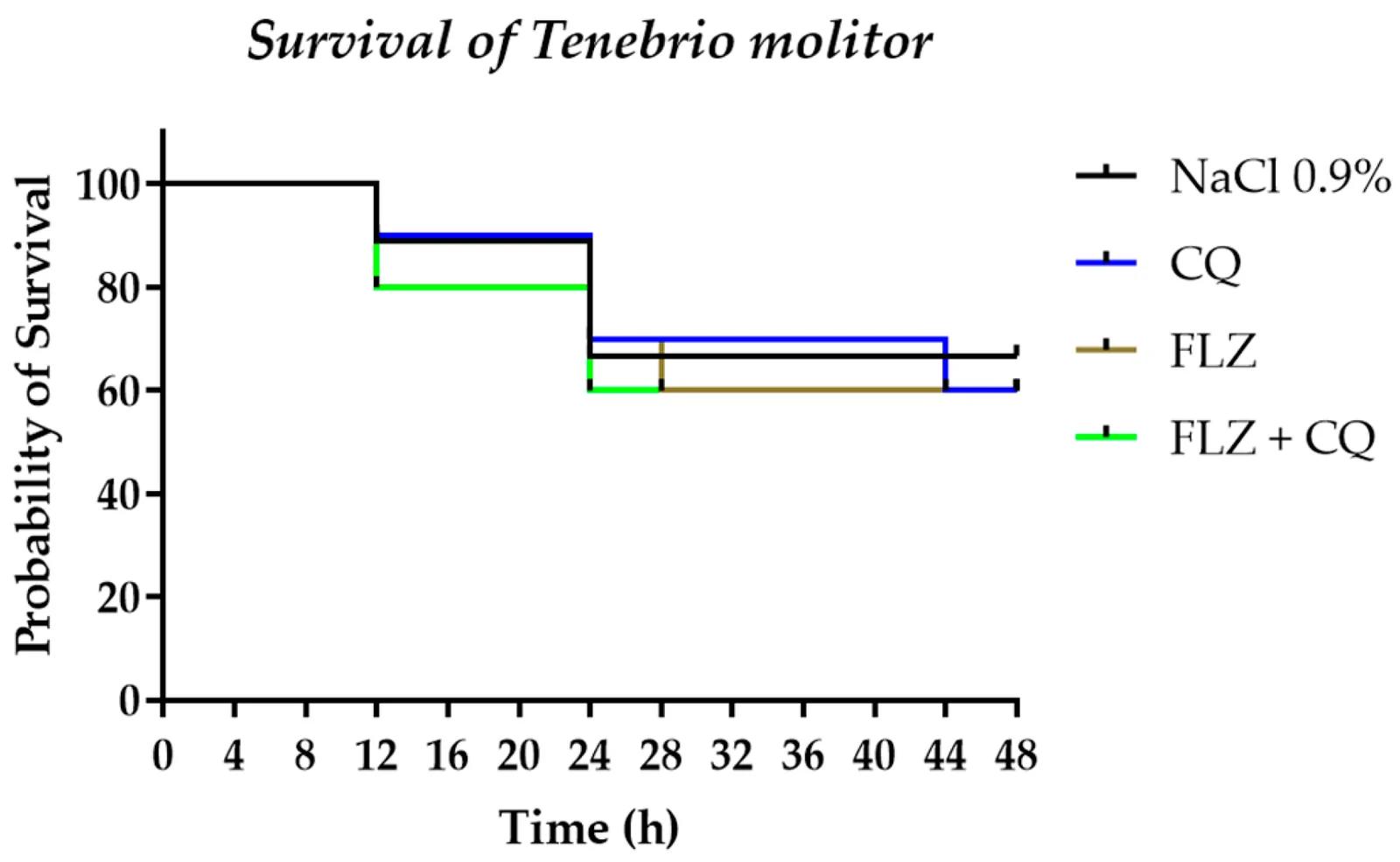
*Tenebrio molitor* larvae exposed to FLZ, CQ, and FLZ + CQ, at concentrations of 16, 2, and 0.125 + 0.25 µg·mL^−1^ for 48 h. Negative control: NaCl 0.9%.

Toxicity tests performed on mealworms (*Tenebrio molitor*) show that the larvae treated with the respective drug concentrations, alone or in combination, did not show a statistically significant difference (Figure 9) concerning the negative control (NaCl 0.9%).

## 4. Discussion

Cryptococcal meningitis is one of the main fungal meningitides commonly diagnosed in immunocompromised patients, especially HIV-positive patients. Its virulence characteristics, such as the presence of a capsule, melanin production, immune system evasion by internalization in macrophages, and migration to the central nervous system, restrict the therapeutic arsenal and contribute to high mortality rates [2,43].

To date, treatment for cryptococcal meningitis is limited to the combination of AMB with 5-FC and/or FLZ. The duration of treatment can be longer than two weeks in some cases, and recovery rates are low, especially in severe cases [7,8]. Sub-Saharan Africa is historically considered the place with the highest incidence of cryptococcal infections [44]. A modeling analysis developed in South Africa estimated the total costs generated by patients with cryptococcal meningitis. Patients treated with two weeks of AMB/5-FC cost approximately USD2700, and AMB/FLZ around USD2200 per patient. Annually, these estimated amounts can reach costs of over 9 million dollars [45]. Thus, the present study aimed to use an alternative combination of gold-standard drugs (AMB and FLZ) and reposition previously reported drugs with antifungal activity, such as CQ and 8HQ.

During the 1950s, clioquinol was widely consumed in Japan in high doses and frequently indicated for therapy for parasitic infections. In 1956, numerous cases of blindness began to be diagnosed (myelo-optic neuropathy syndrome or SMON), which were associated with toxicity from ingestion. After this negative visibility, its marketing was discontinued. However, in the 1990s, clioquinol was studied again and showed promising results in the treatment of Alzheimer’s disease with controlled doses, without adverse effects [46].

Studies have already evaluated the antifungal potential of 8HQ for yeasts, showing low MIC rates [23,26,27,28,29,30,31,32,47,48,49,50] consistent with those found in our study. However, the combination of CQ and 8HQ with FLZ and AMB has not yet been reported in the literature for *Cryptococcus* spp., even if the association of both is recurrent for other antifungal agents, such as terbinafine (TRB) [51], posaconazole [52], echinocandins [53] and non-antifungal drugs [28].

The combinations of FLZ with CQ/8HQ analyzed by the Bliss independence model showed the most significant regions of synergism for both strains, H99 and MYA4093. These combinations reduced the FLZ concentrations (2.67 and 3.39 µg·mL^−1^ of FLZ alone for H99 and MYA4093, respectively) to 0.125 µg·mL^−1^ when administered in combination with 0.25 µg·mL^−1^ of CQ, achieving inhibition of more than 90% of the fungal growth.

Even though the combination of AMB and 8HQ showed significant synergism for MYA 4093 (*C. gattii*), the synergism for H99 (*C. neoformans* var. *grubii*) was smaller. This reduced effect could limit its use in treatment. The heterogeneity of inhibition between *C. neoformans* var. *grubii* and *C. gattii* can be observed for other combinations (CQ vs. 8HQ, AMB vs. CQ, FLZ vs. CQ), although the differences are much less pronounced. This decrease in susceptibility may be related to differences in capsule sizes [54]. Another possibility is that interspecies differences result in different susceptibility profiles due to their metabolic routes and cell wall compositions [55].

Studies have already investigated the main mechanisms of action of 8HQ and derivatives, such as CQ, for fungi. Pippi et al. [56] studied the mechanisms of action of 8HQ derivatives, including CQ, on *Candida albicans*, finding that sulfonic quinolone derivatives and CQ cannot directly bind ergosterol to pore formation. However, the cell extravasation assay shows the loss of nucleic acid through the plasma membrane, indicating irreversible damage only for the sulfonic quinoline derivatives. The opposite occurred with CQ, suggesting that its action would not be directly related to plasma membrane damage.

When we correlate the findings of Pippi et al. [56] with the results reported by Helsel et al. [23], the mechanism of action of 8HQ appears to exhibit ionophoric behavior. This property could explain the cell extravasation observed by Pippi et al. [56], which occurs without direct binding to ergosterol, and the chelating activity of CQ, since its binding to copper added to the culture medium cancels its antifungal activity against *C. neoformans*. Metals such as iron, zinc, and copper are essential for several vital biological functions, including energy production, signal transduction, oxidative stress resistance, and melanin synthesis [57].

The hypothesis about the disturbance in the metal balance in fungal cell death caused by CQ, 8HQ, and derivatives [23,48] supports the synergism found in our study and the inhibition of melanin production by CQ alone and in combination with FLZ. Results found by Kim et al. [58] also corroborate this hypothesis, since, in their study, *C. neoformans* knockout strains encoding the ferroxidase (*CFO1*) and iron permease (*CFT1*) components’ high-affinity reductive iron uptake pathway increase the susceptibility to azole antifungals. Their study found a decrease in the intracellular iron content in cfo1Δ strains, in addition to the influence of metal on numerous metabolic processes, including cellular respiration (electron transport chain dependent on specific iron-containing enzymes).

To our knowledge, the experiment conducted in this study to detect antifungal compounds in RPMI medium after fungal culture using UV-Vis spectroscopy has not been previously reported. The results (Table 1) align with previous reports describing the chelating activity of CQ [59,60] and were expected given its lipophilicity and probable intracellular migration. A similar pattern was anticipated for FLZ, whose mechanism of action involves polar interactions with the carboxylate groups of the heme moiety of CYP51, thereby inhibiting the conversion of lanosterol to ergosterol [61,62]. The reduced extracellular availability of CQ may therefore result from altered lipophilicity following chelation with intracellular metals. Li et al. [59] have demonstrated a higher accumulation of metals in the plasma membrane fraction compared to the cytosolic fraction in *S. cerevisiae*, corroborating our findings.

Analysis of the FLZ–CQ combination in the LB218 isolate (Figure S16), which displays reduced susceptibility to FLZ, revealed a significant synergistic interaction (synergy score = 46 ± 2, *p* < 0.01). These findings highlight a potentially promising therapeutic strategy for cryptococcosis caused by fluconazole–less-susceptible isolates. Notably, Kim et al. [58] reported *Erg11* overexpression in mutant strains relative to the wild type and demonstrated that impaired iron uptake by fungal cells may attenuate the development of azole resistance. This effect is likely mediated by the heme-binding protein Dap-1, whose interaction with heme is critical for *Erg11* activation and, consequently, for ergosterol biosynthesis. Consistently, cells lacking Dap-1 accumulate the *Erg11* substrate and display heightened susceptibility to *Erg11* inhibitors such as FLZ [63].

On the other hand, the combination of FLZ and AMB, one of the most used in the treatment of cryptococcal meningitis, had the lowest synergism score of all combinations. These results are likely due to the mechanisms of action of both drugs when interdependently sharing the target. FLZ acts on the ergosterol biosynthesis route, suppressing its production and decreasing the amount available in the cell. AMB has, as a mechanism, the binding of its molecules to ergosterol, forming pores that lead to the extravasation of cytoplasmic content. Therefore, FLZ may decrease the main action target of AMB, which in turn has difficulty finding enough ergosterol to bind, and they end up indirectly competing for the action target [64].

The low synergism between FLZ and AMB observed in vitro corroborates the clinical outcome, since the associations used in the treatment of cryptococcal meningitis present high mortality rates [43]. In addition, the combination of FLZ and AMB presents a region of antagonism that must be considered carefully as a possible therapeutic failure. Low synergism scores were also found for CQ and 8HQ combination. This low synergism may reflect a problem related to a similar or dependent mechanism of action (interaction with microelements), as well as the combination of FLZ and AMB.

Another positive point for the combination of FLZ and CQ is that the high synergism is maintained for the other strains and isolates tested. CQ was widely studied for the treatment of neurodegenerative diseases [13,65,66]. In a phase 2 clinical trial study, the plasma concentrations of CQ reached up to 7.6 µg·mL^−1^ (fifteen-fold higher than the CQ concentrations in our combination with FLZ) for patients with Alzheimer’s disease receiving daily oral doses of 750 mg. No severe side effects were found, and only one patient had optic neuropathy [13]. Cytotoxicity tests did not demonstrate a reduction in cell viability in concentrations 4 and 32 times higher than those found with synergism for CQ and FLZ, respectively. Furthermore, the HET-CAM and *Tenebrio molitor* tests developed in this study showed that the concentrations tested did not indicate irritation or acute toxicity, suggesting that the combination of FLZ and CQ represents a useful alternative to be explored as a new form of treatment for cryptococcosis.

Numerous articles in the literature, and already cited here in Refs. [29,51,52,53,67], use FICI as a tool to determine the interaction between antifungals and works on the Loewe additivity model. We must be careful when obtaining the interaction between antifungals. The combination assay must be assessed with adequate methodology and statistical analysis of combinations for a good interpretation of the interactions. The improper use of data can modify the synergism/antagonism interaction profile [68]. Medicines whose mechanism of action is different when used in combination have a multiplicative form of interaction (Bliss Independence model). In contrast, drugs with equivalent mechanisms of action can have their activities summed using the Loewe additivity model [69].

Another problem in using FICI for drug interaction studies is the visual reading of the results when considering synergistic points with 100% inhibition. This subjective reading interferes with the accurate quantification and statistical analysis of effects. The levels of synergistic, antagonistic, or indifferent interactions compared to untreated growth control are impossible to determine because an inhibition higher than 100% does not exist. Using the Bliss independence model through the Combenefit software, spectrophotometric readings based on the percentage of growth (IC_50_) and the number of replicates provided precise results in our study. This methodology allows for the measurement of the range of the synergism and the best synergistic region with statistical significance. Furthermore, this means of analysis ensures the repeatability of results.

## 5. Conclusions

The limited number of antifungals to cryptococcosis treatment has remained a challenge for decades. Given the difficulty in finding new molecules, the repositioning and combination of antifungal drugs represent a viable alternative to therapy for difficult-to-treat infections with high mortality rates, such as cryptococcosis. Our results show that the combinations of FLZ and CQ/8HQ presented regions of synergism, with scores up to three-fold higher than the scores obtained by the drugs FLZ and AMB. In addition, the combination of FLZ and CQ was shown to act against the inhibition of melanin production, without showing toxic/irritant effects, at the concentrations tested in the chosen alternative models. The association between molecules containing different mechanisms of action allows for the greater sensitization of fungal cells, culminating in a reduction in the minimum inhibitory concentrations necessary for cell death and consequently reducing the probability of toxic effects. Our findings represent a substantial multi-target strategy to be considered in future animal model trials for the treatment of cryptococcal meningitis, considering the association between metal-chelating molecules (8HQ and CQ) associated with ergosterol-depleting antifungals in the fungal cell membrane.

## Figures and Tables

**Table 1:** Design of experiments used for the calibration of the multivariate method.

Run	FLZ (%)	8HQ/CQ (%)	FLZ (µg·mL^−1^)	8HQ/CQ (µg·mL^−1^)
1	1	0	32	0
2	0	1	0	8
3	0.5	0.5	16	4
4	0.75	0.25	24	2
5	0.25	0.75	8	6
6	0.9	0.1	28.8	0.8
7	0.1	0.9	3.2	7.2
8	0.95	0.05	30.4	0.4
9	0.05	0.95	1.6	7.6
10	0.99	0.01	31.68	0.08
11	0.01	0.99	0.32	7.92
12	0.5	0	16	0
13	0	0.5	0	4
14	0.6	0.4	19.2	3.2
15	0.4	0.6	12.8	4.8

FLZ: fluconazole; 8HQ: 8-hydroxyquinoline; CQ: clioquinol; (µg·mL^−1^).

**Table 2:** Inhibitory concentration (IC_50_ and IC_90_) in µg·mL^−1^ of *Cryptococcus* spp. strains for drugs alone.

Strains	FLZ	AMB	8HQ		CQ	
	IC_50_/R^2^	IC_90_/R^2^	IC_50_/R^2^	IC_90_/R^2^	IC_50_/R^2^	IC_90_/R^2^
H99	2.67/0.97	0.40/0.95	0.75/0.95	1.19/0.93	0.3/0.98	0.38/0.99
MYA4093	3.39/0.95	0.36/0.95	0.57/0.92	2.08/0.95	0.32/0.97	0.42/0.98
LB218	5.53/0.95	0.3/0.95	0.33/0.90	0.43/0.91	0.62/0.96	0.82/0.98
LB619	1.52/0.9	0.31/0.97	2.27/0.88	2.42/92	0.47/0.92	0.64/0.93
LB141	2.83/0.81	0.45/0.91	2.23/0.95	2.41/0.96	0.3/0.99	0.5/0.99
B3501	1.09/0.93	0.19/0.97	0.29/0.91	0.47/0.94	0.26/0.93	0.39/0.95
ATCC 32045	1.15/0.94	0.15/0.91	1.31/0.96	1.87/97	0.38/0.95	0.48/0.95

R^2^: determination coefficient; IC_50_: inhibitory concentration of 50%; IC_90_: inhibitory concentration of 90%; FLZ: fluconazole; AMB: amphotericin B; 8HQ: 8-hydroxyquinoline; CQ: clioquinol.

**Table 3:** Analysis of urea conversion and inhibition of urease production of CQ, FLZ, and FLZ + CQ (µg·mL^−1^) in the H99 strain after six days of cultivation, with the reading performed at 492 nm.

Urea Conversion											
Drug [ ]	CQ			Drug [ ]	FLZ			Drug [ ]	FLZ/CQ		
8	N/C	N/C	N/C	64	N/C	N/C	N/C	4/8	N/C	N/C	N/C
4	N/C	N/C	N/C	32	N/C	N/C	N/C	2/4	N/C	N/C	N/C
2	N/C	N/C	N/C	16	N/C	N/C	N/C	1/2	N/C	N/C	N/C
1	N/C	N/C	N/C	8	N/C	C	C	0.5/1	C	C	C
0.5	N/C	N/C	N/C	4	C	C	C	0.25/0.5	C	C	C
0.25	N/C	C	C	2	C	C	C	0.125/0.25	C	C	C
0.125	C	C	C	1	C	C	C	0.065/0.125	C	C	C
0.065	C	C	C	0.5	C	C	C	0.031/0.065	C	C	C
**Urea Inhibition**											
**Drug [ ]**	**CQ**			**Drug [ ]**	**FLZ**			**Drug [ ]**	**FLZ/CQ**		
1	I	I	I	8	N/I	N/I	N/I	0.5/1	N/I	N/I	N/I
0.5	I	I	I	4	N/I	N/I	N/I	0.25/0.5	N/I	N/I	N/I
0.25	N/I	N/I	N/I	2	N/I	N/I	N/I	0.125/0.25	N/I	N/I	N/I
0.125	N/I	N/I	N/I	1	N/I	N/I	N/I	0.065/0.125	N/I	N/I	N/I
0.065	N/I	N/I	N/I	0.5	N/I	N/I	N/I	0.031/0.065	N/I	N/I	N/I

N/C: no conversion; C: conversion; I: inhibition; N/I: no inhibition; [ ]: concentration (µg·mL^−1^).

## Data Availability

The original contributions presented in this study are included in the article/Supplementary Material. Further inquiries can be directed to the corresponding author.

## Supplementary Materials

The following supporting information can be downloaded at: https://www.mdpi.com/article/10.3390/microorganisms14081654/s1, Figure S1: Scheme of the checkerboard test performed in the evaluation of combinations for H99 (*C. neoformans* var. *grubii*) and MYA 4093 (*C. gattii*); Table S1: Analysis of urea conversion and inhibition of urease production by MYA 4093 strain after six days of culture and reading performed at 492 nm.; Figure S2: Fluconazole (FLZ) and clioquinol (CQ) dose-response curves, score matrix and contour plot for the FLZ vs. CQ strain H99 (*C. neoformans* var. *grubii*); Figure S3: Fluconazole (FLZ) and clioquinol (CQ) dose-response curves, score matrix and contour plot for the FLZ vs. CQ strain MYA 4093 (*C. gattii*); Figure S4: Fluconazole (FLZ) and 8-hydroxyquinoline (8HQ) dose-response curves, score matrix, and contour plot for the FLZ vs. 8HQ of the H99 strain (*C. neoformans* var. *grubii*); Figure S5: Fluconazole (FLZ) and 8-hydroxyquinoline (8HQ) dose-response curves, score matrix, and contour plot for the FLZ vs. 8HQ of strain MYA 4093 (*C. gattii*); Figure S6: Fluconazole (FLZ) and amphotericin B (AMB) dose-response curves, score matrix, and contour plot for the FLZ vs. AMB from the H99 strain (*C. neoformans* var. *grubii*); Figure S7: Fluconazole (FLZ) and amphotericin B (AMB) dose-response curves, score matrix and contour plot for the FLZ vs. AMB strain MYA 4093 (*C. gattii*); Figure S8: Amphotericin B (AMB) and clioquinol (CQ) dose-response curves, score matrix, and contour plot for the AMB vs. CQ of the H99 strain (*C. neoformans* var. *grubii*); Figure S9: Amphotericin B (AMB) and clioquinol (CQ) dose-response curves, score matrix, and contour plot for the AMB vs. CQ of the MYA 4093 strain (*C. gattii*); Figure S10: Clioquinol (CQ) and 8-hydroxyquinoline (8HQ) dose-response curves, score matrix, and contour plot for the CQ vs. 8HQ of the H99 strain (*C. neoformans* var. *grubii*); Figure S11: Clioquinol (CQ) and 8-hydroxyquinoline (8HQ) dose-response curves, score matrix and contour plot for the CQ vs. 8HQ of strain MYA 4093 (*C. gattii*); Figure S12: Amphotericin B (AMB) and 8-hydroxyquinoline (8HQ) dose-response curves, score matrix, and contour plot for the AMB vs. 8HQ of the H99 strain (*C. neoformans* var. *grubii*); Figure S13: Amphotericin B (AMB) and 8-hydroxyquinoline (8HQ) dose-response curves, score matrix, and contour plot for the AMB vs. 8HQ of strain MYA 4093 (*C. gattii*); Figure S14: Fluconazole (FLZ) and clioquinol (CQ) dose-response curves, score matrix and contour plot for the FLZ vs. CQ strain B3501 (*C. neoformans*); Figure S15: Fluconazole (FLZ) and Clioquinol (CQ) dose-response curves, score matrix and contour plot for the FLZ vs CQ combination of the clinical isolate LB619 (*C. neoformans* var. *grubii*); Figure S16: Fluconazole (FLZ) and Clioquinol (CQ) dose-response curves, score matrix and contour plot for the FLZ vs CQ combination of the clinical isolate LB218 (*C. gattii*); Figure S17: Fluconazole (FLZ) and clioquinol (CQ) dose-response curves, score matrix and contour plot for the FLZ vs CQ combination of the ATCC 32045 (*C. neoformans*) strain; Figure S18: Fluconazole (FLZ) and clioquinol (CQ) dose-response curves, score matrix and contour plot for the FLZ vs CQ combination of the clinical isolate LB141 (*C. neoformans*); Figure S19: UV/Vis spectra (220–400 nm) for FLZ and 8HQ in combination or alone in the RPMI 1640 and samples of *C. neoformans* and *C. gattii* after 48 h of FLZ and 8HQ cultivation in the PRMI medium. The tested concentrations of FLZ and 8HQ in culture with *C. neoformans* and *C. gattii* were 2 and 8 µg·mL^−1^, respectively; 8HQ in 4 µg·mL^−1^ culture medium; FLZ + 8HQ in 1.6 and 7.6 µg·mL^−1^ culture medium, respectively. FLZ in 16 µg·mL^−1^ culture medium. Negative control: RPMI 1640.
