# Supplementary material for: Synergic Effect of Fluconazole and Quinoline Derivatives Combination Against Cryptococcus spp., Mechanisms of Action and Toxicity
Source: Microorganisms. 2026 Jul 29;14(8):1654. doi: 10.3390/microorganisms14081654 (PMC13515043; doi:10.3390/microorganisms14081654)
Supplement: Supplementary file 1 [file microorganisms-14-01654-s001.zip › microorganisms-3966541-supplementary.pdf]

SUPPORTING INFORMATION

Synergic effect of fluconazole and quinoline derivatives combination against Cryptococcus spp., mechanisms of action and toxicity

Luana Candice Genz Bazana[1,2*], Ânderson Ramos Carvalho[1,2], Alexandre Meneghello Fuentefria[1,2].

[1] Laboratório de Pesquisa em Micologia Aplicada, Porto Alegre, RS, Brazil.

[2] Programa de Pós-Graduação em Ciências Farmacêuticas, Universidade Federal do Rio Grande do Sul (UFRGS), Porto Alegre, RS, Brazil.

*** Corresponding Author:**

Luana Candice Genz Bazana,

Laboratory of Applied Mycology, College of Pharmacy, Annex II,

Federal University of Rio Grande do Sul,

São Luís, 154, ZIP code: 90470-440, Porto Alegre – RS, Brazil.

Phone: +5551980479067

e-mail: luanacgb93@hotmail.com

Table S1. Analysis of urea conversion and inhibition of urease production by MYA 4093 strain after six days of culture and reading performed at 492 nm.

MYA 4093												
	**Drug [ ]**	**CQ**			**Drug [ ]**	**FLZ**			**Drug [ ]**	**FLZ/CQ**		
**Urea conversion**	8	C	C	C	64	C	C	C	4/8	C	C	N/C
	4	N/C	N/C	N/C	32	N/C	N/C	N/C	2/4	N/C	N/C	N/C
	2	N/C	N/C	N/C	16	N/C	N/C	N/C	1/2	N/C	N/C	N/C
	1	N/C	N/C	N/C	8	C	C*	C*	0.5/1	N/C	N/C	C
	0.5	N/C	N/C	N/C	4	C	C	C	0.25/0.5	C	C	C
	0.25	N/C	N/C	C	2	C	C	C	0.125/0.25	C	C	C
	0.125	C	C	C	1	C	C	C	0.065/0.125	C	C	C
	0.065	C	C	C	0.5	C	C	C	0.031/0.065	C	C	C
	Drug [ ]	**CQ**			**Drug [ ]**	**FLZ**			**Drug [ ]**	**FLZ/CQ**		
**Urea inhibition**	1	I	I	I	8	N/I	I*	I*	0.5/1	I	I	N/I
	0.5	I	I	I	4	N/I	N/I	N/I	0.25/0.5	N/I	N/I	N/I
	0.25	I	I	N/I	2	N/I	N/I	N/I	0.125/0.25	N/I	N/I	N/I
	0.125	N/I	N/I	N/I	1	N/I	N/I	N/I	0.065/0.125	N/I	N/I	N/I
	0.065	N/I	N/I	N/I	0.5	N/I	N/I	N/I	0.031/0.065	N/I	N/I	N/I

N/C: no conversion. C: conversion. I: inhibition. N/I non-inhibition.*Indication of inhibition of urease production.

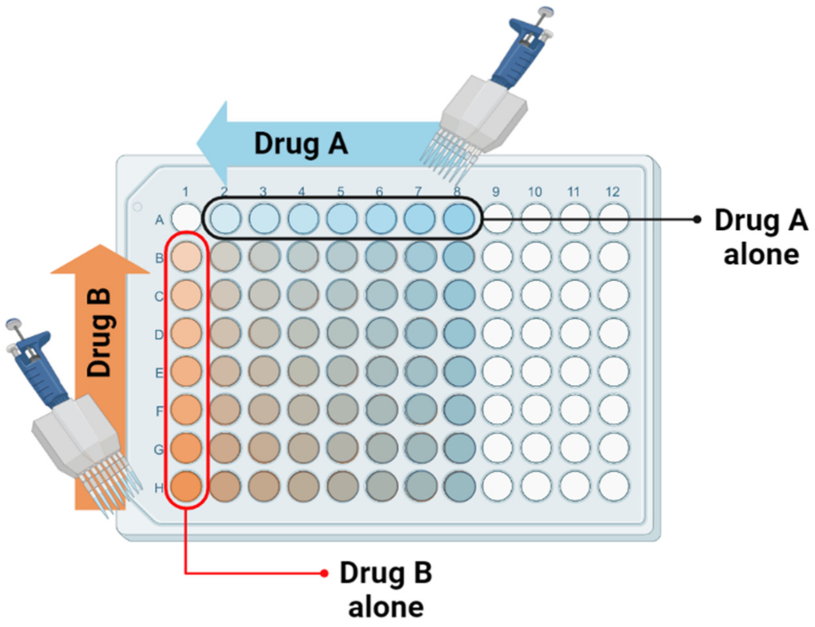

Figure S1. Scheme of the checkerboard test performed in the evaluation of combinations for H99 (*C. neoformans* var*. grubii*) and MYA 4093 (*C. gattii*).

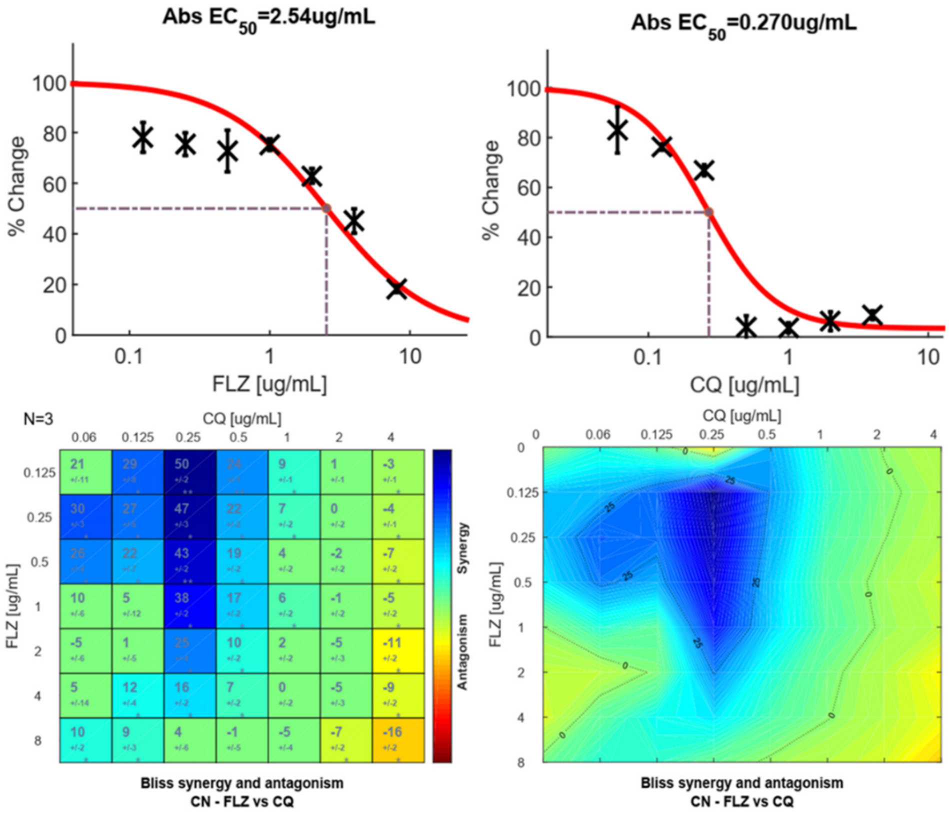

Figure S2. Fluconazole (FLZ) and clioquinol (CQ) dose-response curves, score matrix and contour plot for the FLZ vs. CQ strain H99 (*C. neoformans* var. *grubii*).

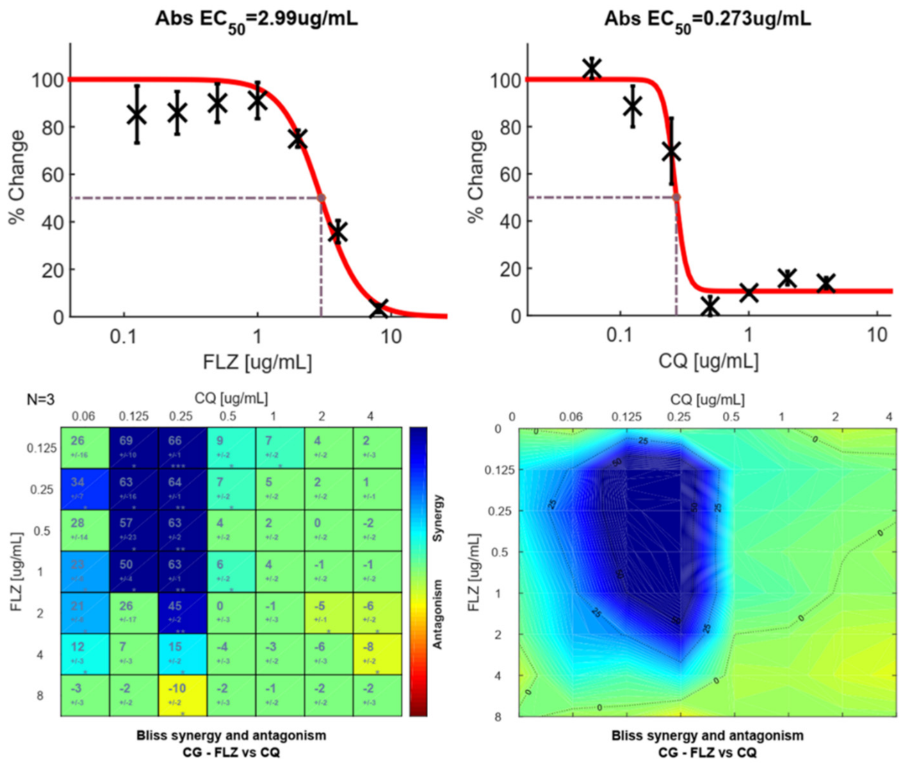

Figure S3. Fluconazole (FLZ) and clioquinol (CQ) dose-response curves, score matrix and contour plot for the FLZ vs. CQ strain MYA 4093 (*C. gattii*).

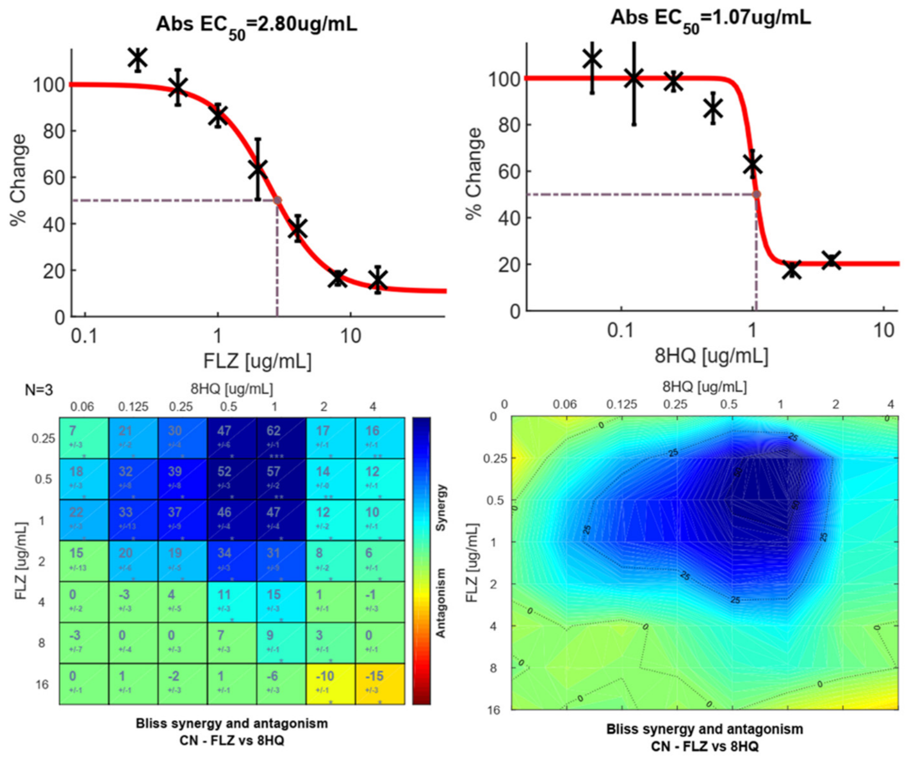

Figure S4. Fluconazole (FLZ) and 8-hydroxyquinoline (8HQ) dose-response curves, score matrix, and contour plot for the FLZ vs. 8HQ of the H99 strain (*C. neoformans* var. *grubii*).

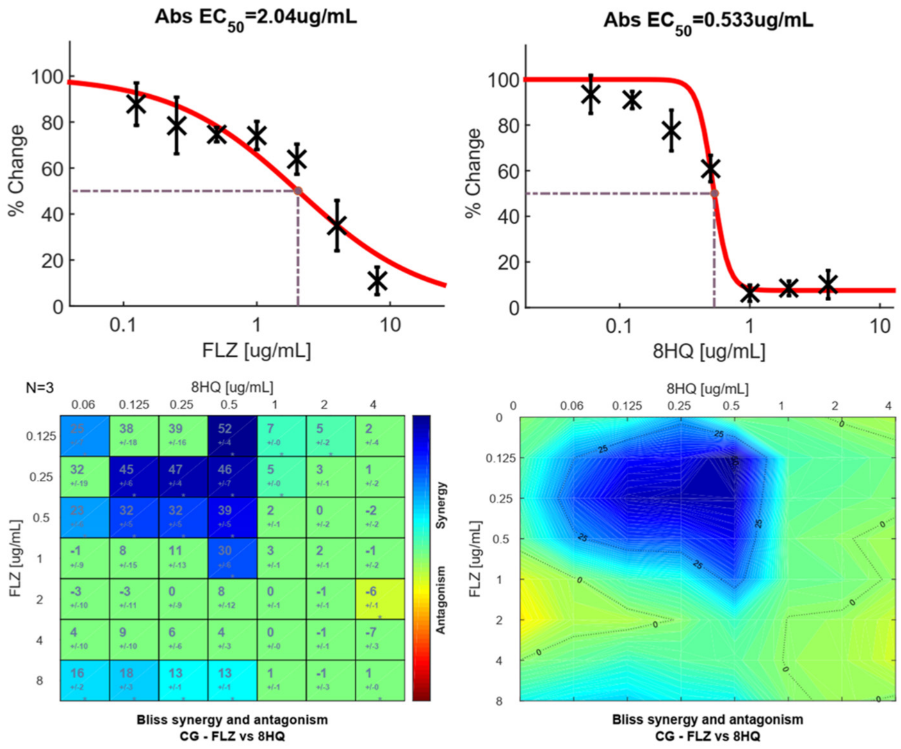

Figure S5. Fluconazole (FLZ) and 8-hydroxyquinoline (8HQ) dose-response curves, score matrix, and contour plot for the FLZ vs. 8HQ of strain MYA 4093 (*C. gattii)*.

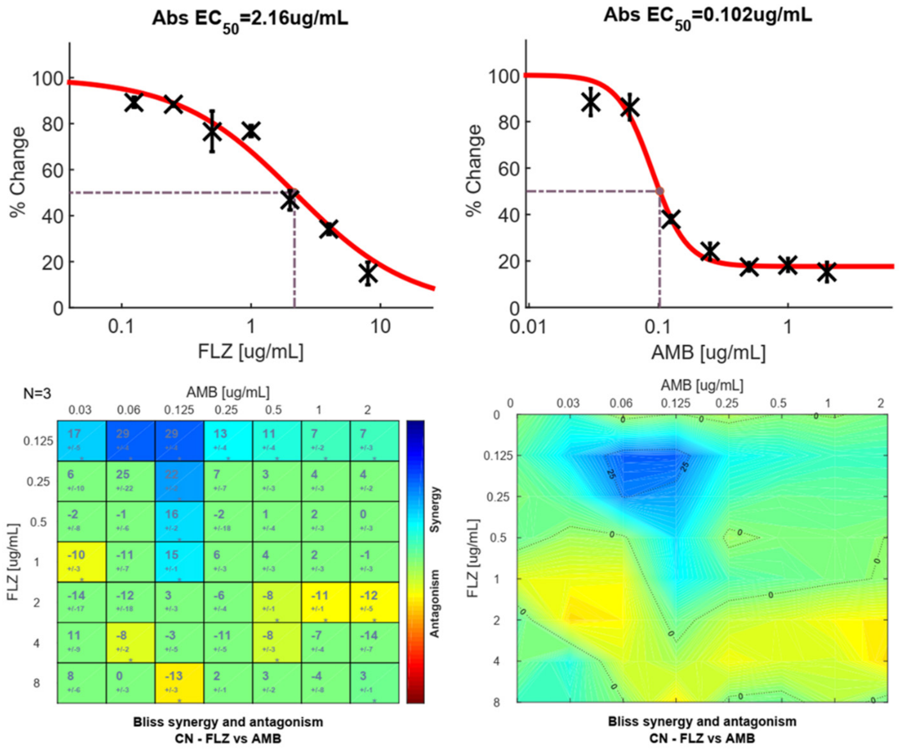

Figure S6. Fluconazole (FLZ) and amphotericin B (AMB) dose-response curves, score matrix, and contour plot for the FLZ vs. AMB from the H99 strain (*C. neoformans* var. *grubii*).

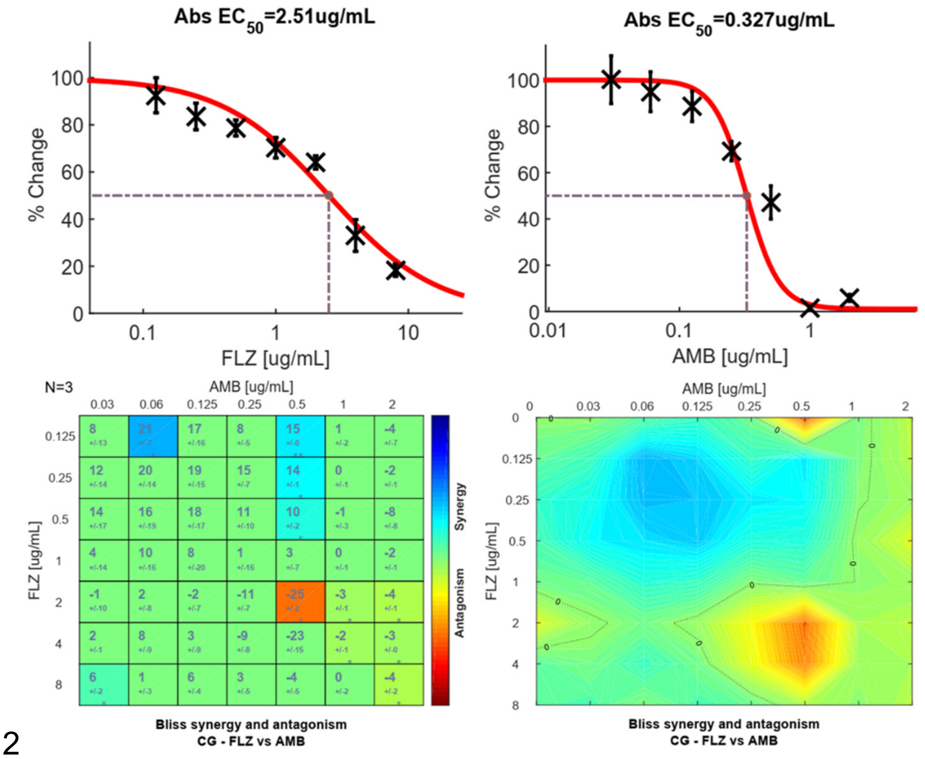

12

Figure S7. Fluconazole (FLZ) and amphotericin B (AMB) dose-response curves, score matrix and contour plot for the FLZ vs. AMB strain MYA 4093 (*C. gattii*).

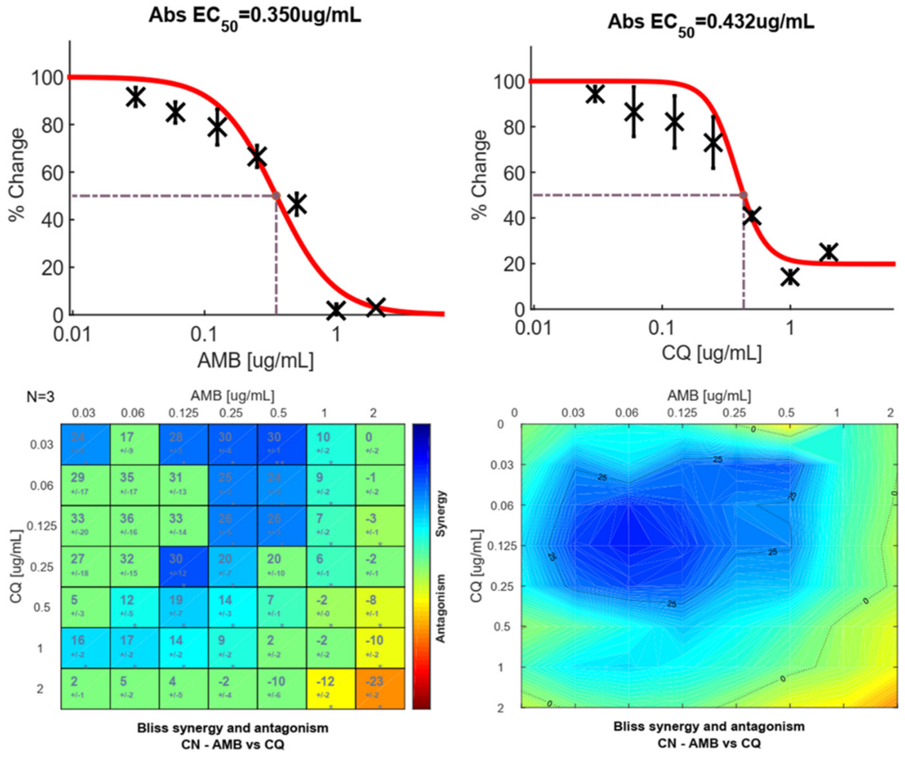

Figure S8. Amphotericin B (AMB) and clioquinol (CQ) dose-response curves, score matrix, and contour plot for the AMB vs. CQ of the H99 strain (*C. neoformans* var. *grubii*).

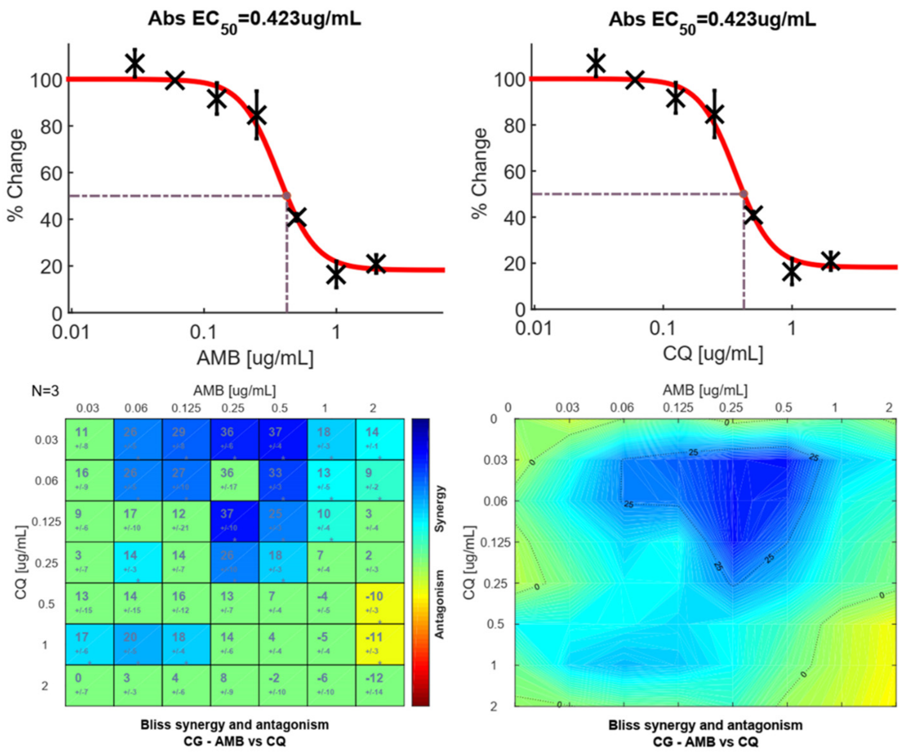

Figure S9. Amphotericin B (AMB) and clioquinol (CQ) dose-response curves, score matrix, and contour plot for the AMB vs. CQ of the MYA 4093 strain (*C. gattii*).

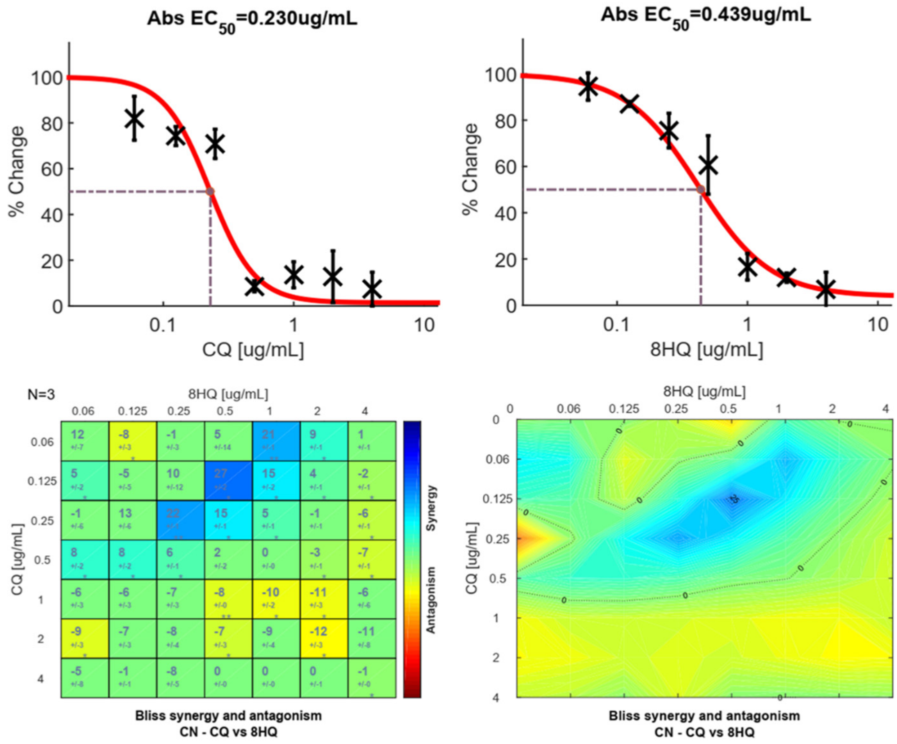

Figure S10. Clioquinol (CQ) and 8-hydroxyquinoline (8HQ) dose-response curves, score matrix, and contour plot for the CQ vs. 8HQ of the H99 strain (*C. neoformans* var. *grubii*).

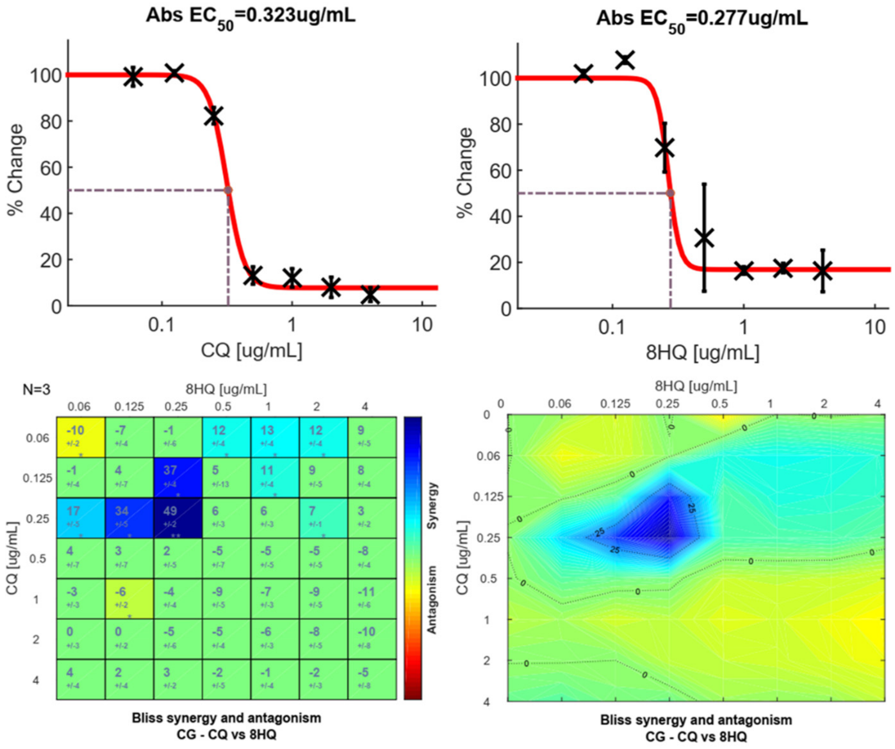

Figure S11. Clioquinol (CQ) and 8-hydroxyquinoline (8HQ) dose-response curves, score matrix and contour plot for the CQ vs. 8HQ of strain MYA 4093 (*C. gattii*).

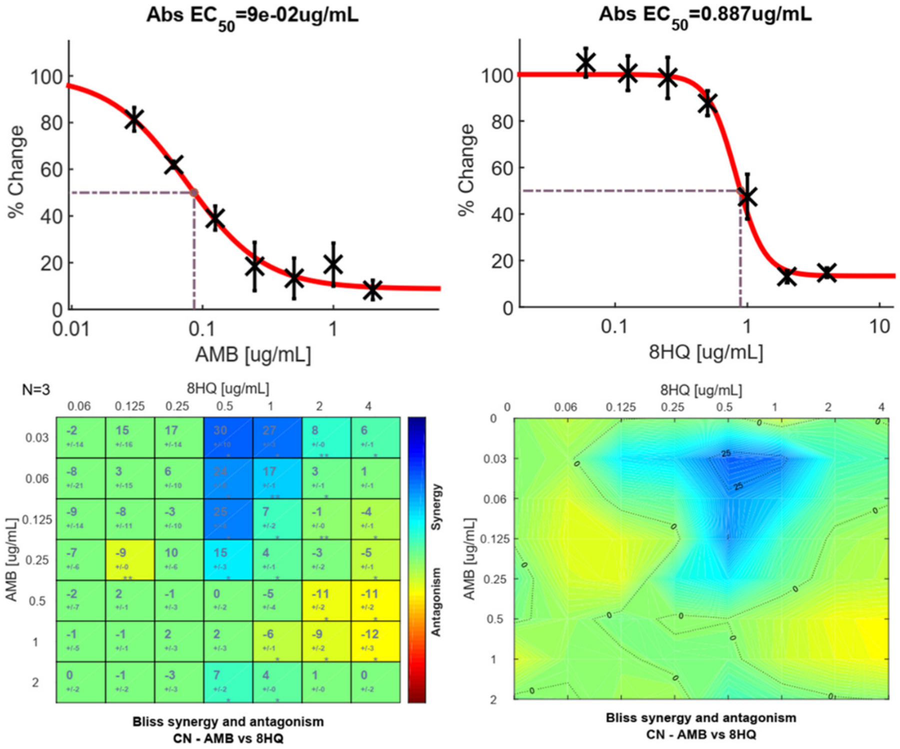

Figure S12. Amphotericin B (AMB) and 8-hydroxyquinoline (8HQ) dose-response curves, score matrix, and contour plot for the AMB vs. 8HQ of the H99 strain (*C. neoformans* var*. grubii*).

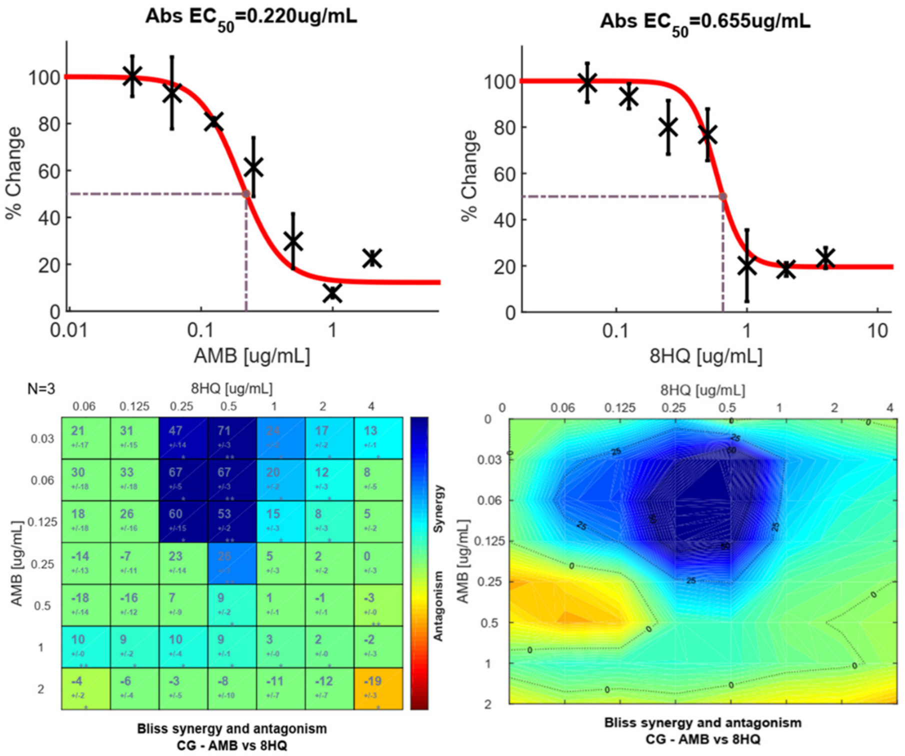

Figure S13. Amphotericin B (AMB) and 8-hydroxyquinoline (8HQ) dose-response curves, score matrix, and contour plot for the AMB vs. 8HQ of strain MYA 4093 (*C. gattii*).

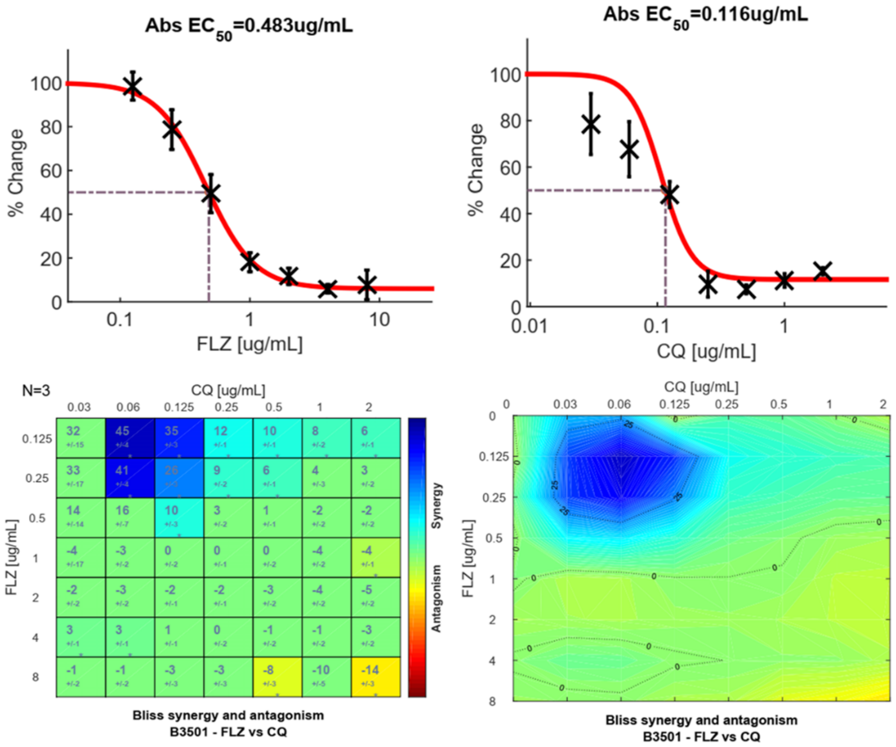

Figure S14. Fluconazole (FLZ) and clioquinol (CQ) dose-response curves, score matrix and contour plot for the FLZ vs. CQ strain B3501 (*C. neoformans*).

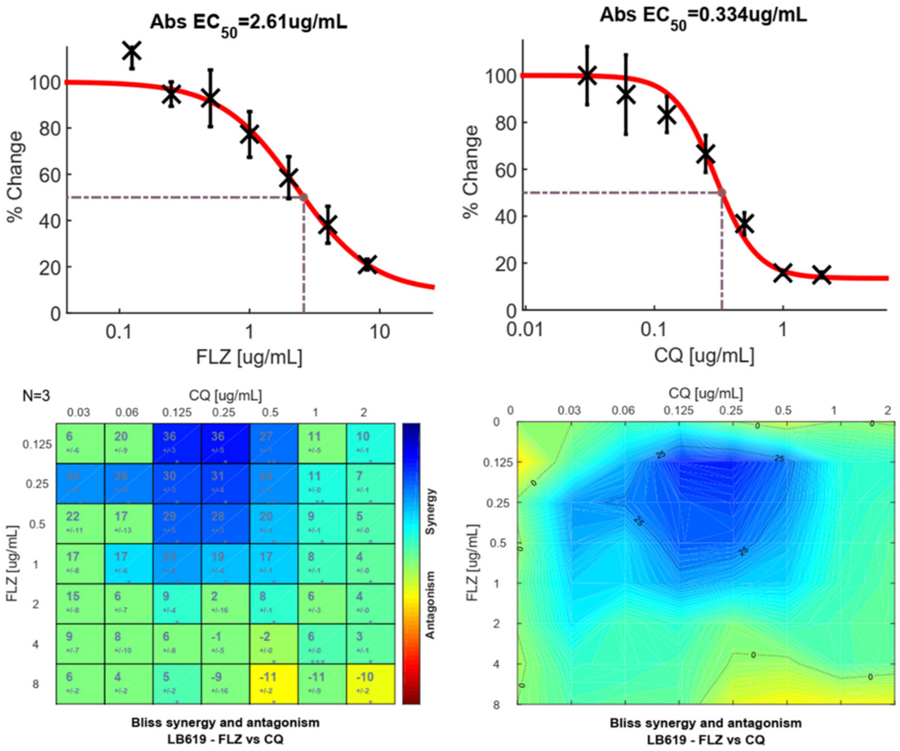

Figure S15. Fluconazole (FLZ) and Clioquinol (CQ) dose-response curves, score matrix and contour plot for the FLZ vs CQ combination of the clinical isolate LB619 (*C. neoformans* var. *grubii*).

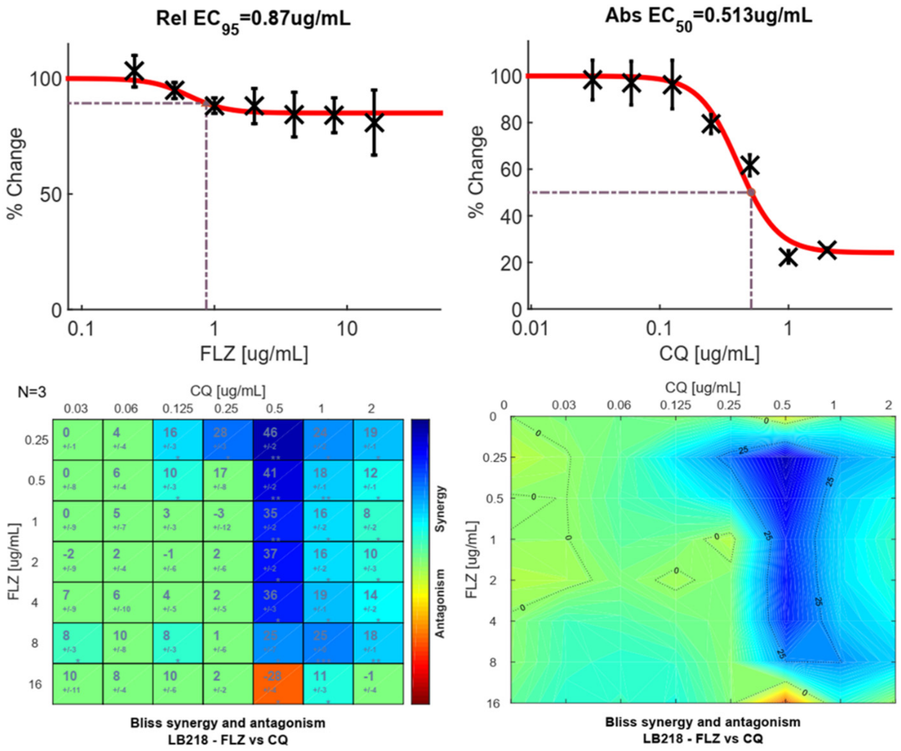

Figure S16. Fluconazole (FLZ) and Clioquinol (CQ) dose-response curves, score matrix and contour plot for the FLZ vs CQ combination of the clinical isolate LB218 (*C. gattii*).

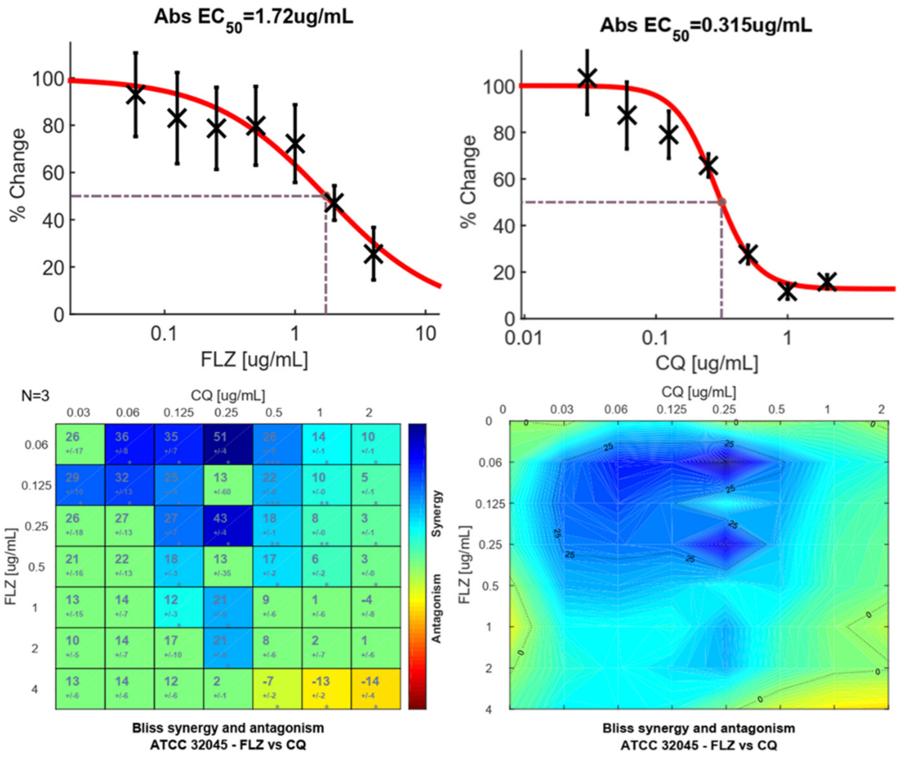

Figure S17. Fluconazole (FLZ) and clioquinol (CQ) dose-response curves, score matrix and contour plot for the FLZ vs CQ combination of the ATCC 32045 (*C. neoformans*) strain.

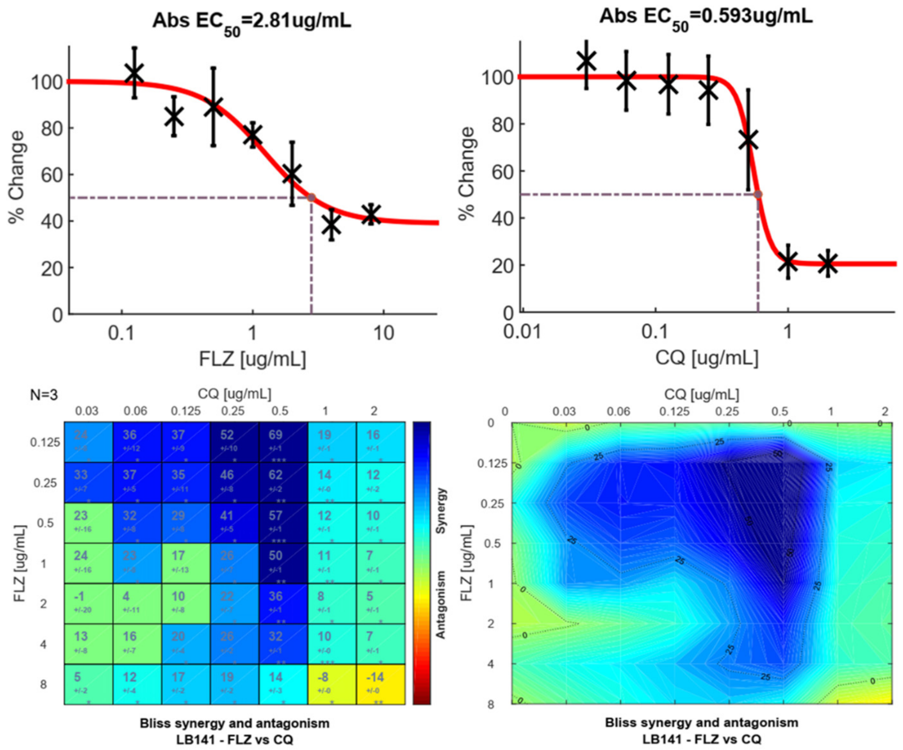

Figure S18. Fluconazole (FLZ) and clioquinol (CQ) dose-response curves, score matrix and contour plot for the FLZ vs CQ combination of the clinical isolate LB141 (*C. neoformans*).

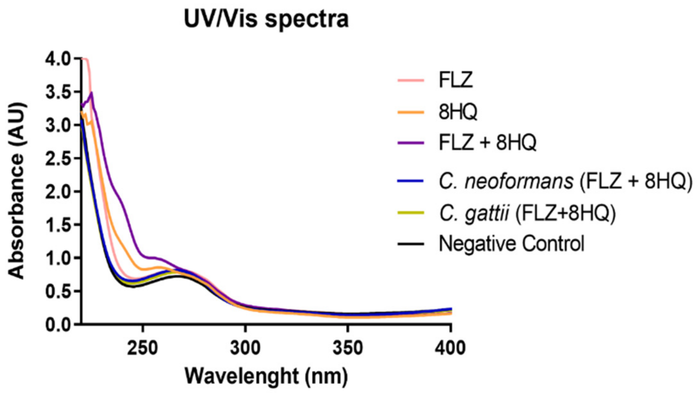

Figure S19. UV/Vis spectra (220-400 nm) for FLZ and 8HQ in combination or alone in the RPMI 1640 and samples of *C. neoformans* and *C. gattii* after 48 hours of FLZ and 8HQ cultivation in the PRMI medium. The tested concentrations of FLZ and 8HQ in culture with *C. neoformans* and *C. gattii* were 2 and 8 $\mu g.mL^{-1}$, respectively; 8HQ in 4 $\mu g.mL^{-1}$ culture medium; FLZ + 8HQ in 1.6 and 7.6 $\mu g.mL^{-1}$ culture medium, respectively. FLZ in 16 $\mu g.mL^{-1}$ culture medium. Negative control: RPMI 1640.
